# SIGLEC12 mediates plasma membrane rupture during necroptotic cell death

**DOI:** 10.1038/s41586-025-09741-1

**Published:** 2025-11-12

**Authors:** Hyunjin Noh, Zeena Hashem, Elena Boms, Ayaz Najafov

**Affiliations:** Department of Internal Medicine, UT Southwestern Medical Center, Dallas, TX USA

**Keywords:** Cell death, Proteases, Extracellular signalling molecules, Signal transduction

## Abstract

Necroptosis is a form of lytic cell death that is overactivated during infections and in inflammatory pathologies^1^. NINJ1 was recently found to be a mediator of plasma membrane rupture (PMR) during pyroptosis, toxin-induced necrosis, apoptosis, and ferroptosis^2,3^, but the mediator of PMR during necroptotic cell death remained unknown. Here, using a CRISPR–Cas9-based genome-wide knockout approach, we identify SIGLEC12 as a key mediator of necroptosis downstream of MLKL at the PMR step. Cells with knockdown or knockout of *SIGLEC12* are defective in necroptosis-induced PMR and demonstrate ballooning morphology. During necroptosis, SIGLEC12 undergoes dephosphorylation, interacts with MLKL, forms cytosolic puncta and assembles into fibrils. Notably, SIGLEC12 is cleaved by TMPRSS4 during necroptosis to produce a 20-kDa fragment highly homologous to NINJ1, and this cleavage event is required and sufficient to induce PMR during necroptosis. A SIGLEC12 variant associated with cancer (Ser458Phe) and a variant found in the general human population (Arg528Trp) attenuate SIGLEC12 cleavage by TMPRSS4. Knockout of *Siglec12* in mouse cells does not affect PMR, suggesting a species-specific role. Our identification of SIGLEC12 as a mediator of PMR expands our understanding of how programmed necrosis is executed and offers new approaches for targeting this proinflammatory form of cell death in human diseases.

## Abstract (web summary)

CRISPR–Cas9-based genome-wide screening shows that SIGLEC12 mediates plasma membrane rupture in cells undergoing necroptosis, possibly through a human-specific mechanism, indicating its potential as a therapeutic target.

## Main

Necroptosis is mediated by a signalling cascade culminating in the oligomerization of a pseudokinase called MLKL, which forms pore-like structures that allow water influx, leading to PMR^4^. Execution of PMR during pyroptosis, toxin-induced necrosis, apoptosis and ferroptosis requires NINJ1, but this transmembrane protein is not required for PMR during necroptosis downstream of MLKL^2,5,6^. Thus, our mechanistic understanding of the molecular events occurring downstream of MLKL is incomplete.

## SIGLEC12 mediates necroptosis downstream of MLKL

**Fig. 1:**
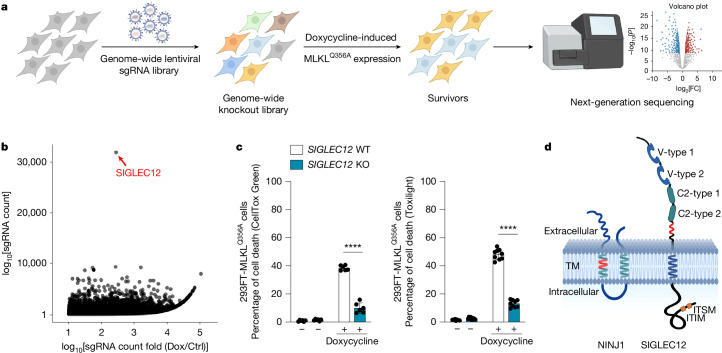
SIGLEC12 is a mediator of necroptosis. **a**, Outline of the genome-wide CRISPR–Cas9-based knockout screen performed in HEK293-FlpIn-Trex (293FT)-MLKL^Q356A^ cells to identify mediators of necroptosis. FC, fold change. **b**, Volcano plot summarizing the screening results. **c**, 293FT-MLKL^Q356A^ cells with or without *SIGLEC12* knocked out were treated with 0.1 μg ml^−1^ doxycycline for 24 h, and cell death was quantified using CellToxGreen or Toxilight (*P* = 3.54 × 10^−7^ (left), 9.01 × 10^−13^ (right)). Data are plotted as the mean ± s.d. (*n* = 6 (left) or *n* = 9 (right)) from three independent experiments. **d**, Predicted structures of NINJ1 and SIGLEC12. The NISI (NINJ1/SIGLEC12 homology) motif is indicated in red. Statistical analyses were performed using two-tailed *t*-test; ****P < 0.0001. V-type, V-type immunoglobulin; C2-type, C2-type immunoglobulin; ITIM, immunoreceptor tyrosine-based inhibitory motif; ITSM, immunoreceptor tyrosine-based switch motif; TM, transmembrane. Panels **a** and **d** were created using BioRender (https://biorender.com).

**Extended Data Fig. 1:**
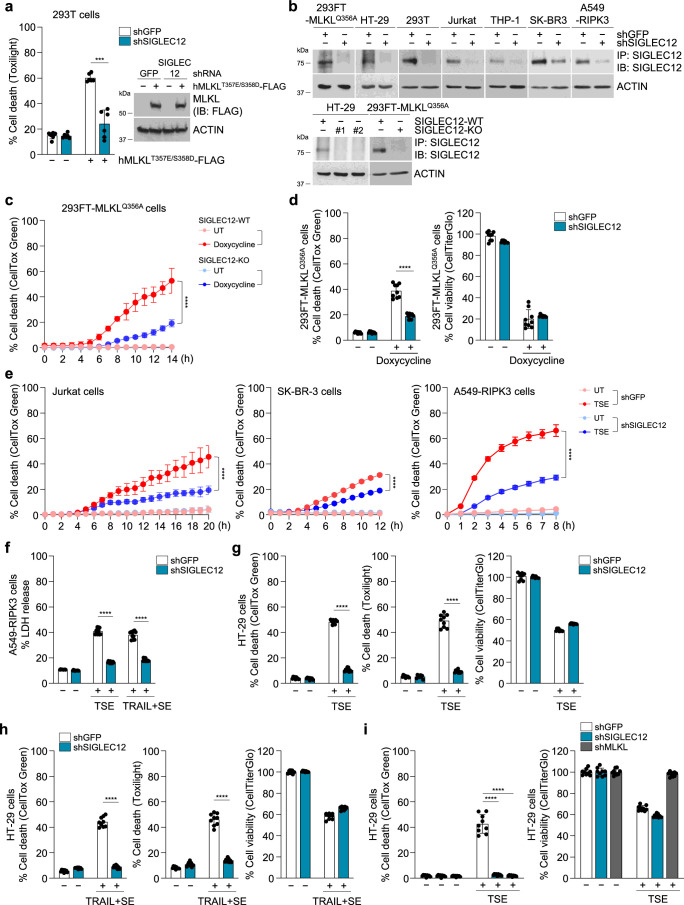
Plasma membrane rupture downstream of MLKL is mediated by SIGLEC12. **a**, 293 T cells were transfected with a constitutively active human MLKL^T357E/S358D^ mutant for 24 h. Cell death was quantified using Toxilight (p-value = 2.67 × 10^−4^), and protein expression levels were assessed using immunoblotting for FLAG-tagged MLKL. **b**, Stable shRNA-mediated knockdowns were validated in the indicated cells by immunoprecipitation with anti-SIGLEC12 antibody, followed by immunoblotting for SIGLEC12. **c**, 293FT-MLKL^Q356A^ cells with indicated knockouts were treated with 0.1 μg/ml doxycycline, and cell death was quantified using CellToxGreen. Green fluorescence intensity was measured every hour for 14 h using Incucyte S3 (p-value < 1 × 10^−15^). **d**, 293FT-MLKL^Q356A^ cells with indicated stable shRNA-mediated knockdowns were treated with 0.1 μg/ml doxycycline for 24 h, and cell death (CellToxGreen) or cell viability (CellTiterGlo) was quantified (p-value = 8.73 × 10^−7^). **e-i**, Jurkat, SK-BR-3, A549-RIPK3 cells (**e, f**) or HT-29 cells (**g-i**) with indicated shRNA-mediated stable knockdowns were treated with TSE or TRAIL + SE for 8 h and cell death (CellToxGreen, Toxilight, or LDH release) or cell viability (CellTiterGlo) were quantified (p-value = <1 × 10^−15^ (**e**), 3.2 × 10^−14^, 2.73 × 10^−12^ (**f**), <1 × 10^−15^, 4.4 × 10^−9^ (**g**), 2.96 × 10^−10^, 5.6 × 10^−9^ (**h**), and 1.7 × 10^−7^, 1.54 × 10^−7^ (**i**)). Green fluorescence intensity was measured every hour for the indicated times using Incucyte S3. Data were plotted as the mean ± s.d., representative of n = 4 (**c** and **e**, left and middle), n = 6 (**a** and **e**, right), n = 9 (**d**, **g-i**) or n = 12 (**f**); three independent experiments. Statistical analyses were performed using two-tailed *t*-test and two-way ANOVA; ***p < 0.001, ****p < 0.0001. Data are representative of three independent experiments (**a**, **b**).

To determine mechanisms of necroptosis execution downstream of MLKL, we used a doxycycline-inducible expression system in HEK293 cells (FlpIn-Trex)^7^ to express a constitutively active form of MLKL (MLKL^Q356A^)^8,9^ on the background of a genome-wide CRISPR–Cas9-based knockout library^10^ (Fig. 1a). Strikingly, the outlier hit whose knockout protected cells from cell death induced by MLKL^Q356A^ expression was a transmembrane protein called SIGLEC12 (Fig. 1b). This observation was confirmed in our follow-up experiments using further single-guide RNA (sgRNA) sequences (Fig. 1c), with a similar observation made for the constitutively active MLKL^T357E/S358D^ phosphomimetic^11^ (Extended Data Fig. 1a).

SIGLEC12 (sialic-acid-binding Ig-like lectin 12) is a member of the SIGLEC family of immunoglobulin-like lectins, which are primarily expressed on immune cells and play key parts in the regulation of immune responses through recognition of sialic-acid-containing glycans^12–14^. SIGLEC12 has two V-type sialic-acid-binding immunoglobulin domains and two C2-type immunoglobulin domains in its extracellular region, as well as an immunoreceptor tyrosine-based inhibitory motif and an immunoreceptor tyrosine-based switch motif in its intracellular region^14^ (Fig. 1d).

Unlike other members of the SIGLEC family, human SIGLEC12 has lost its sialic-acid-binding activity owing to several mutations in its V-type immunoglobulin domains^12,14,15^. SIGLEC12 is expressed in the gastrointestinal tract, epithelial cells and macrophages, as well as certain cancers, in which it may contribute to tumour progression^13–15^. The inability of SIGLEC12 to bind to sialic acid in humans suggests a potential species-specific role in the homeostasis of biological processes native to human physiology.

## SIGLEC12 mediates PMR downstream of MLKL

**Fig. 2:**
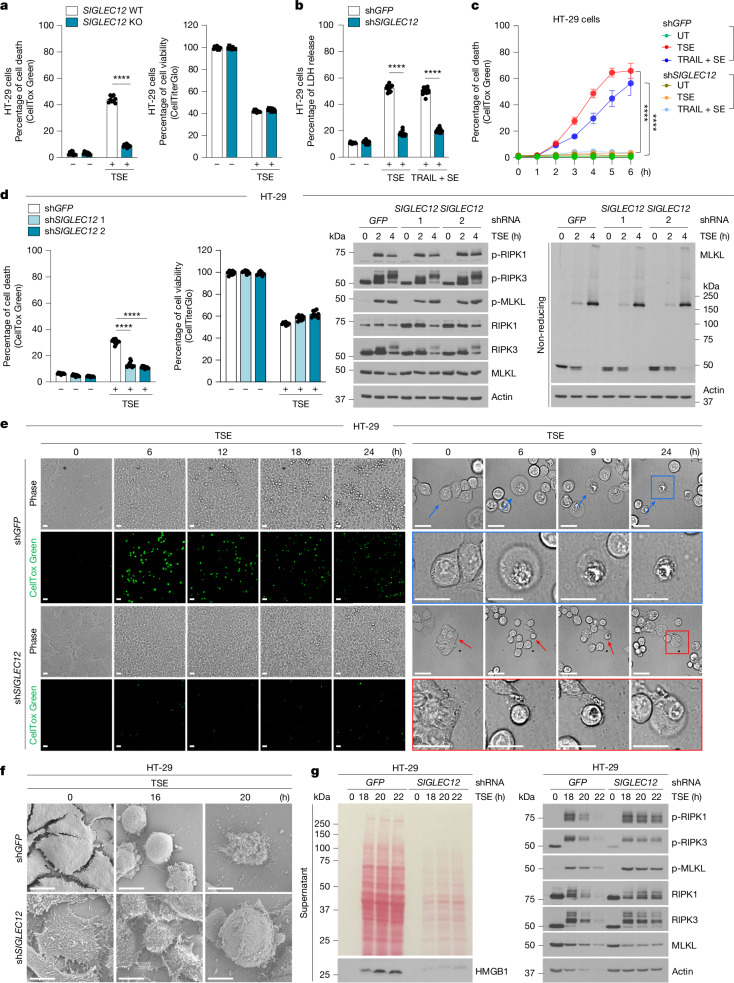
PMR downstream of MLKL is mediated by SIGLEC12. **a**–**d**, HT**-**29 cells with the indicated knockouts (**a**) or knockdowns (**b**–**d**) were treated with TSE (30 ng ml^−1^ hTNF, 0.2 µM SM-164, 5 µM emricasan) or TRAIL + SE (50 ng ml^−1^, 0.2 µM SM-164, 5 µM emricasan) for 8 h, and cell death or cell viability was quantified using the indicated assays (*P* = 1.39 × 10^−13^ (**a**), *P* < 1 × 10^−15^ (**b**), *P *< 1 × 10^−15^ (**c**) and *P* = 6.82 × 10^−12^, *P *= 1.23 × 10^−11^ (**d**)). Cell lysates were analysed using reducing or non-reducing gel electrophoresis with the indicated antibodies. **e**, HT-29 cells with indicated stable short hairpin RNA (shRNA)-mediated knockdowns were treated with TSE for the indicated times, and cell death was assessed using CellToxGreen and time-lapse fluorescence confocal microscopy. **f**, As in **e**, except cell morphology was visualized by scanning electron microscopy. **g**, As in **e**, except the release of HMGB1 was analysed by immunoblotting. Data are plotted as the mean ± s.d., representative of *n* = 4 (**c**), *n* = 9 (**a**,**d**) or *n* = 12 (**b**); three independent experiments. Statistical analyses were performed using two-tailed *t*-tests and two-way analyses of variance; ****P < 0.0001. Data are representative of three independent experiments (**d**, middle and right, **g**). Scale bars, 25 μm (**e**), 10 μm (**f**).

**Extended Data Fig. 2:**
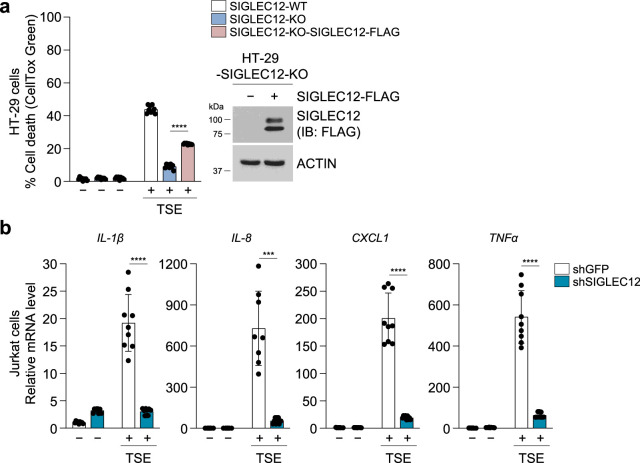
SIGLEC12-dependent plasma membrane rupture downstream of MLKL and cytokine production during necroptosis. **a**, Cell death was assessed by CellToxGreen fluorescence in HT-29-SIGLEC12-WT, HT-29-SIGLEC12-KO, and HT-29-SIGLEC12-KO-SIGLEC12-FLAG stable cell lines treated with TSE for 8 h (p-value = 1.48 × 10^−10^). **b**, Jurkat cells with indicated stable shRNA-mediated knockdowns were treated with TSE for 7 h. *IL-1β*, *IL-8*, *CXCL1*, and *TNF* mRNA levels were measured by qRT-PCR (p-value = 1.21 × 10^−5^, 2 × 10^−4^, 2.24 × 10^−6^, 2.96 × 10^−10^, 3.15 × 10^−6^). Data were plotted as the mean ± s.d., representative of n = 9 (**a, b**); three independent experiments. Statistical analyses were performed using two-tailed *t*-test and two-way ANOVA; ***p < 0.001, ****p < 0.0001. Data are representative of three independent experiments (**a**).

Consistent with the screen finding, *SIGLEC12* knockout and knockdown protected cells from necroptotic cell death induced by (1) expression of the constitutively active form of MLKL (MLKL^Q356A^), (2) the necroptosis-inducing TSE (TNF, SM-164, emricasan) cocktail or (3) the necroptosis-inducing TRAIL + SE (TRAIL, SM-164, emricasan) cocktail (Fig. 2a–d and Extended Data Figs. 1b–i and 2a). Notably, SIGLEC12 loss protected cells from becoming positive for CellToxGreen (a membrane-impermeant fluorescent DNA probe that stains cells only when plasma membrane is ruptured), as well as protecting them from LDH release, but not from cell death, as judged by loss of ATP levels using a CellTiter-Glo assay, suggesting that the role of SIGLEC12 is likely to involve mediation of PMR (Fig. 2a–e (left), Extended Data Figs. 1b–i and 2a and Supplementary Videos 1–4). Loss of SIGLEC12 did not alter activation of the necroptosis mediators RIPK1, RIPK3 and MLKL, as judged by their autophosphorylation markers and MLKL oligomerization (Fig. 2d). The inability of SIGLEC12-deficient cells to rupture plasma membrane during necroptosis resulted in a ‘bubble’ phenotype observed by light and electron microscopy (Figs. 2e (right) and 2f and Supplementary Videos 5 and 6). Finally, the defective PMR in SIGLEC12-deficient cells resulted in diminished release of cytosolic proteins and HMGB1 (Fig. 2g) and production of the proinflammatory cytokines IL-1β, IL-8, CXCL1 and TNF^16^ (Extended Data Fig. 2b).

**Extended Data Fig. 3:**
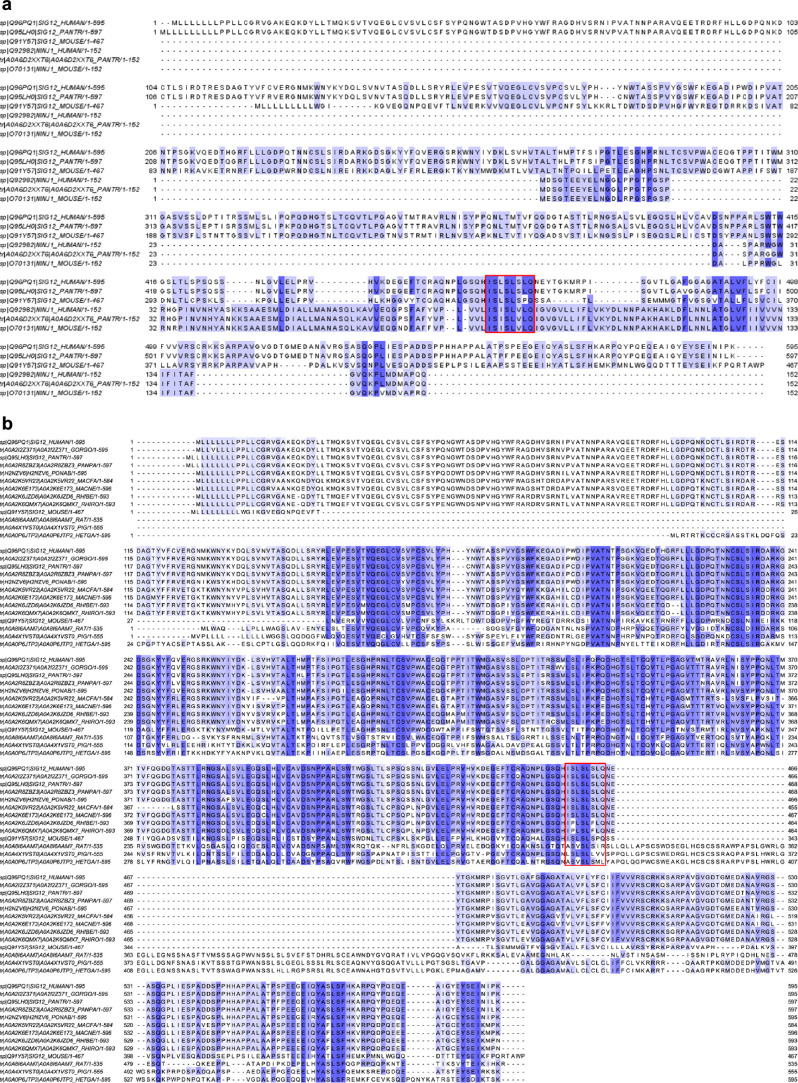
Amino acid sequence alignment of SIGLEC12 and NINJ1 across species. **a**, Alignment for SIGLEC12 versus NINJ1 for the indicated species. **b**, Alignment for SIGLEC12 for the indicated species. The NISI (NINJ1/SIGLEC12 homology motif) motifs are highlighted with red boxes.

**Extended Data Fig. 4:**
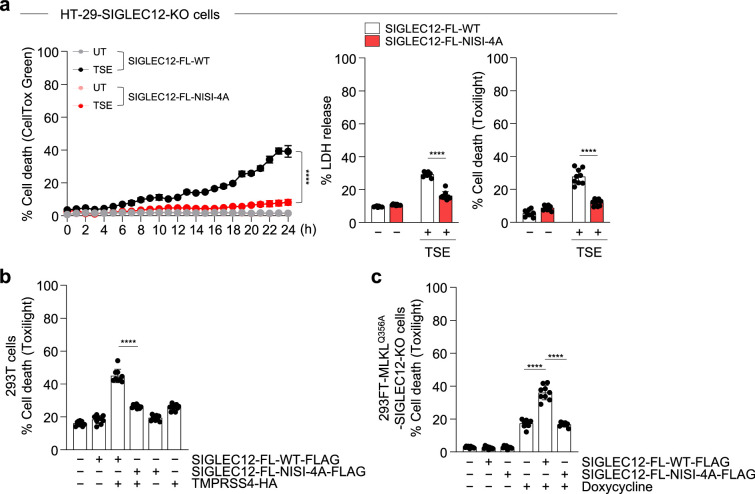
The role of the NISI motif in PMR during necroptosis. **a**, HT-29-SIGLEC12-KO-SIGLEC12-FL-WT or NISI mutant (NISI-4A) stable cell lines were treated with TSE, and cell death was measured by green fluorescence intensity every hour for the indicated times using Incucyte S3 (p-value = <1 × 10^−15^, 5.99 × 10^−9^, 1.53 × 10^−6^). **b, c**, 293 T cells were co-transfected with indicated SIGLEC12-FL-FLAG plasmids ± TMPRSS4-HA for 40 h (**b**). 293FT-MLKL^Q356A^-SIGLEC12-KO cells were transfected with the indicated SIGLEC12-FL-FLAG plasmids and treated with 0.1 μg/ml doxycycline for 24 h to induce the expression of MLKL-Q356A (**c**). Cell death was measured using the Toxilight assay (p-value = 2.91 × 10^−7^ (**b**) and 8.26 × 10^−8^, 3.74 × 10^−7^ (**c**)). Data were plotted as the mean ± s.d., representative of n = 3 (**a**, left) or n = 9 (**a**, middle and right, **b** and **c**); three independent experiments. Statistical analyses were performed using two-tailed *t*-test and two-way ANOVA; ****p < 0.0001.

These findings are analogous to those of Kayagaki et al.^2^ with respect to the role of NINJ1 in PMR during pyroptosis, toxin-induced necrosis and apoptosis, suggesting that whereas NINJ1 mediates PMR during pyroptosis and apoptosis, SIGLEC12 mediates PMR during necroptosis. Consistently, we found striking amino acid similarity between SIGLEC12 and NINJ1, indicating possible functional homology (Fig. 1d and Extended Data Fig. 3). Owing to the high degree of similarity, we propose that this sequence be called the NINJ1/SIGLEC12 homology motif or NISI motif. Notably, mutating the first four residues of the SIGLEC12 NISI motif (ISLS) to four alanine residues blocked PMR during necroptosis, confirming the functionality of this motif in SIGLEC12 (Extended Data Fig. 4).

**Extended Data Fig. 5:**
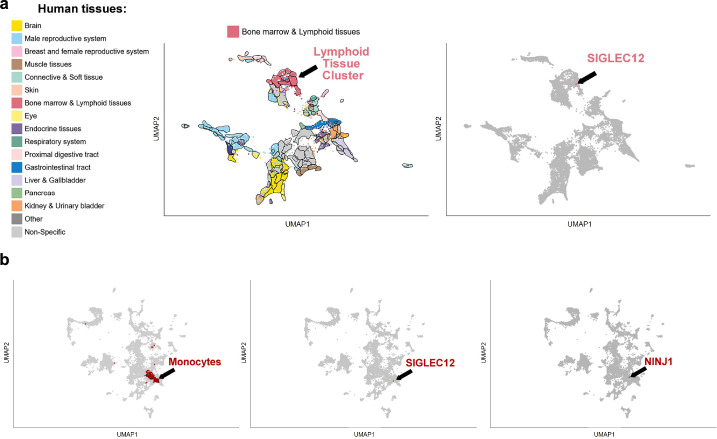
SIGLEC12 expression is enriched in the same clusters as that of NINJ1. **a, b**, The clustering data for the gene expression sets from the deep sequencing of RNA (RNA-seq) from 40 different normal tissue types (https://www.proteinatlas.org/ENSG00000254521-SIGLEC12/tissue) (**a**) and single-cell RNA sequencing (scRNAseq) (**b**) clustering data from 31 human tissue types (https://www.proteinatlas.org/ENSG00000254521-SIGLEC12/single+cell). The data was obtained from the ProteinAtlas.org database, version 22. UMAP plots display gene clusters from Louvain clustering of gene expression across all tissue types or single cell types.

In our analysis of human pan-tissue RNA sequencing (RNA-seq) data curated in ProteinAtlas, *SIGLEC12* expression was enriched in the bone marrow and lymphoid tissue cluster, which contained the gene encoding MLKL and other necroptosis-related genes, including those coding for FasL, TNFR2, TRAIL-R2, the IFNα, IFNβ and IFNγ receptors, TNFAIP3 (A20), CIAP2, CFLAR, PELI1, TBK1 and ZBP1 (Extended Data Fig. 5a). Similarly, cell subtype analysis of human single-cell RNA sequencing (scRNA-seq) data showed that *SIGLEC12* expression was enriched in the monocyte cluster, together with necroptosis-related genes including *TNFRSF1B* (encoding TNFR2), *TNFRSF10B* (encoding TRAIL-R2), *TNFAIP3* (encoding A20), *IFNB1*, *IFNGR2*, *CFLAR*, *PELI1* and *TBK1*, as well as *NINJ1* (Extended Data Fig. 5b). These observations further suggest a functional connection between SIGLEC12 and NINJ1.

## SIGLEC12 is regulated during necroptosis

**Fig. 3:**
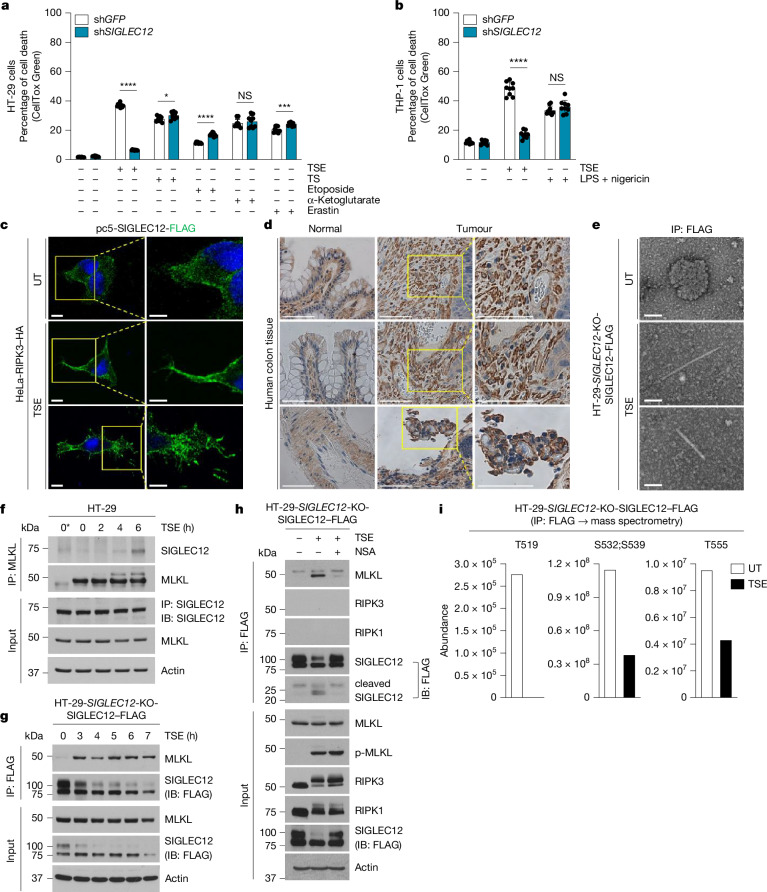
Regulation of SIGLEC12 during necroptosis. **a**,**b**, HT-29 cells (**a**) and THP-1 cells (**b**) with indicated stable shRNA-mediated knockdowns were treated with TSE (necroptosis, 8 h), TS (extrinsic apoptosis, 12 h), etoposide (intrinsic apoptosis, 24 h), α-ketoglutarate (pyroptosis, 24 h), LPS + nigericin (pyroptosis, 8 h) or erastin (ferroptosis, 24 h). Cell death was quantified using the indicated assays (*P* = 3.96 × 10^−13^, *P *= 3.24 × 10^−2^, *P *= 3.83 × 10^−8^, *P *= 0.46, *P *= 3.96 × 10^−4^ (**a**); and *P *= 2.9 × 10^−10^, *P *= 0.196 (**b**)). **c**, HeLa-RIPK3–HA cells were transfected with SIGLEC12–FLAG for 16 h and treated with TSE for 4 h. Cells were analysed by confocal microscopy following immunofluorescence for FLAG. SIGLEC12 was localized to the plasma membrane and cytosolic puncta during necroptosis. **d**, Human normal colon and colon tumour tissue biopsies from patients were stained with SIGLEC12 (*n* = 3). **e**, The HT-29-*SIGLEC12*-KO-SIGLEC12–FLAG stable cell line was treated with TSE, and SIGLEC12–FLAG was immunoprecipitated and analysed by transmission electron microscopy. **f**,**g**, HT-29 cells (**f**) and HT-29-*SIGLEC12*-KO-SIGLEC12–FLAG stable cells (**g**) were treated with TSE for the indicated times. Cell lysates and indicated immunoprecipitation samples were immunoblotted with the indicated antibodies. 0* indicates the IgG control antibody lane. **h**, The HT-29-*SIGLEC12*-KO-SIGLEC12–FLAG stable cell line was treated with TSE for 4 h with or without NSA (2 μM) cotreatment. SIGLEC12–FLAG was immunoprecipitated, eluted with FLAG peptide and analysed by immunoblotting. **i**, The HT-29-*SIGLEC12*-KO-SIGLEC12–FLAG stable cell line was treated with TSE for 4 h, and SIGLEC12–FLAG was immunoprecipitated. Phosphomapping was performed using mass spectrometry. Data are plotted as the mean ± s.d., representative of *n* = 9 (**a**,**b**); three independent experiments. Statistical analyses were performed using two-tailed *t*-tests; NS, not significant; *P < 0.05; ***P < 0.001; ****P < 0.0001. Data are representative of three independent experiments (**f**–**h**). Scale bars, 10 μm (**c**), 100 μm (**d**), 100 nm (**e**).

As NINJ1 was found to be important for PMR in different cell death contexts^2,3,17^ but dispensable for PMR during necroptosis^2,5,6^, we tested whether SIGLEC12 was important for PMR in cell death contexts other than necroptosis. Under the conditions tested, SIGLEC12 was dispensable for PMR during extrinsic apoptosis (TNF + SM-164), intrinsic apoptosis (etoposide), pyroptosis (α-ketoglutarate and LPS + nigericin) and ferroptosis (erastin) (Fig. 3a,b). These results indicated that the role of SIGLEC12 in mediation of PMR could be necroptosis-specific.

**Extended Data Fig. 6:**
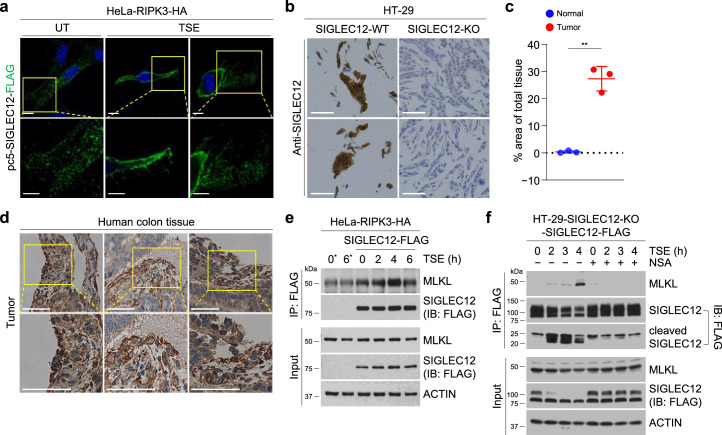
Regulation of SIGLEC12 during necroptosis. **a**, HeLa-RIPK3-HA cells were transfected with SIGLEC12-FLAG for 16 h and treated with TSE for 4 h. Cells were analyzed by confocal microscopy following immunofluorescence for FLAG. SIGLEC12 is localized to the plasma membrane and cytosolic puncta during necroptosis. Scale bars, 10 μm. **b**, Cell plugs for HT-29-SIGLEC12-WT or KO cells were immunostained for endogenous SIGLEC12 and imaged using brightfield microscopy at 20x magnification. Scale bars, 100 μm. **c**, SIGLEC12 IHC signal intensity for slides shown in Fig. 3d was quantified using Fiji/ImageJ (p-value = 8.61×10^−3^). **d**, Human colon tumor tissue biopsies from patients were stained with SIGLEC12 (n = 3). Scale bars, 100 μm. **e**, HeLa-RIPK3-HA cells were transfected with SIGLEC12-FLAG for 16 h and treated with TSE for the indicated times. Cell lysates and indicated immunoprecipitation samples were immunoblotted with the indicated antibodies. **f**, HT-29-SIGLEC12-KO-SIGLEC12-FLAG stable cell line was treated with TSE for the indicated time points with or without NSA co-treatment. SIGLEC12-FLAG was immunoprecipitated and analyzed by immunoblotting. Data were plotted as the mean ± s.d., representative of n = 3 (**c**); three independent experiments. Statistical analyses were performed using two-tailed *t*-test and and two-way ANOVA; **p < 0.01. Data are representative of three independent experiments (**e**, **f**).

Similar to NINJ1 (during pyroptosis^2^), SIGLEC12 formed puncta during necroptosis and translocated to the plasma membrane (Fig. 3c and Extended Data Fig. 6a). Notably, SIGLEC12 formed puncta in human colon tumour biopsies but not normal colon tissue (Fig. 3d and Extended Data Fig. 6b–d), consistent with necroptosis being induced in the intratumour microenvironment^18,19^. Electron microscopy observations showed that SIGLEC12 formed fibrils during necroptosis (Fig. 3e), an assembly feature that was recently shown to be critical for PMR induction by NINJ1 (ref. ^20^). Consistent with its role in mediation of necroptosis downstream of MLKL, SIGLEC12 interacted with MLKL during necroptosis but not with RIPK1 or RIPK3 (Fig. 3f–h and Extended Data Fig. 6e), and this interaction was inhibited by treatment with the MLKL inhibitor necrosulfonamide (NSA) (Fig. 3h and Extended Data Fig. 6f), which blocks binding of MLKL to the plasma membrane^21,22^.

**Extended Data Fig. 7:**
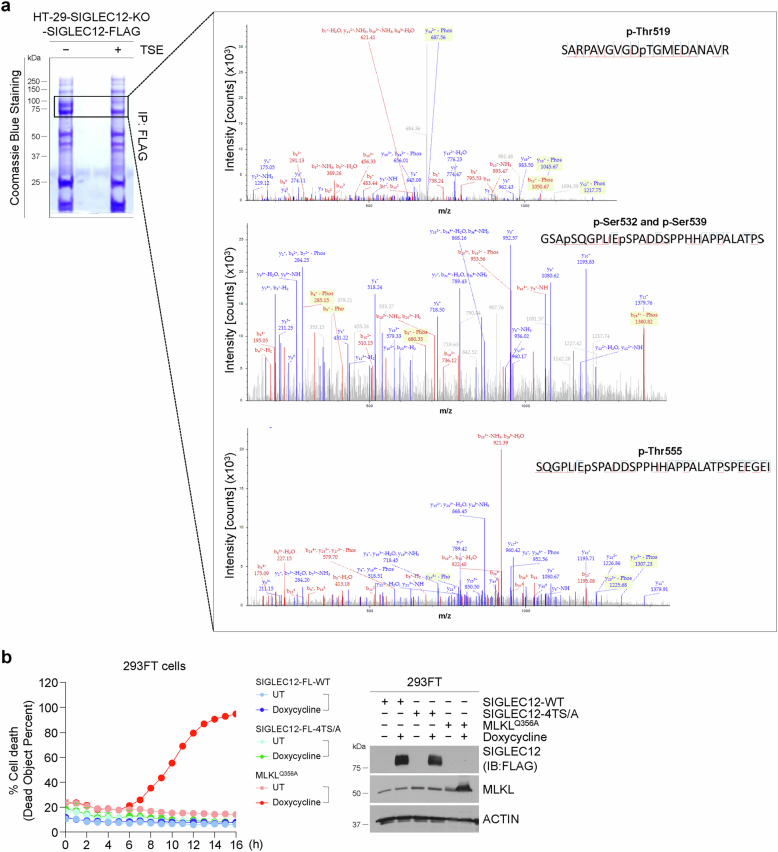
Mass spectrometry-based analysis of SIGLEC12 phosphorylation during necroptosis and its effect of SIGLEC12-mediated PMR. **a**, A large-scale IP-FLAG of SIGLEC12 from HT-29-SIGLEC12-KO-SIGLEC12-FLAG stable cell line following indicated treatments was resolved on SDS-PAGE and stained with Coomassie brilliant blue. Boxes indicate the bands that were analyzed by mass spectrometry to perform phosphomapping. LC-MS/MS ion fragmentation graphs of peptides containing phospho-T519, phospho-S532/S539, and phospho-T555, respectively. pT and pS indicate the phosphorylated residues. **b**, 293FT-SIGLEC12-FL-WT, 293FT-SIGLEC12-FL-4TS/A, and 293FT-MLKL^Q356A^ cells were treated with 0.1 μg/ml doxycycline, and cell death was quantified using Incucyte S3. Data were plotted as the mean ± s.d., representative of n = 4 (**b**); three independent experiments. Data are representative of three independent experiments (**a**, **b**).

Mass spectrometry-based phosphomapping showed that SIGLEC12 was dephosphorylated at Thr519, Ser532;Ser539 and Thr555 during necroptosis (Fig. 3i and Extended Data Fig. 7a). Thr519 and Thr555 are not conserved in rodents, whereas Ser532 is conserved in mouse but not in rat, and Ser539 is conserved in both mouse and rat orthologues (Extended Data Fig. 3b). Mutation of these four residues to phosphorylation-resistant alanine to mimic the dephosphorylated status of SIGLEC12 did not induce PMR (Extended Data Fig. 7b), suggesting that the dephosphorylation event that occurs during necroptosis is not sufficient for activation of the PMR-inducing function of SIGLEC12.

**Extended Data Fig. 8:**
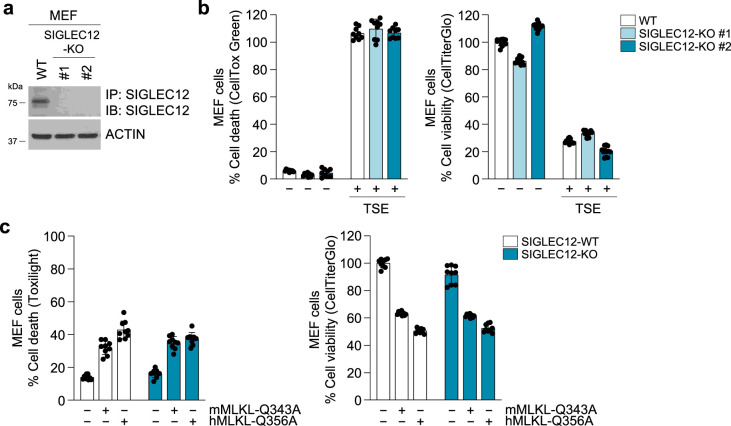
Comparison of human and mouse constitutively active MLKL mutants for their requirements for SIGLEC12 to induce PMR. **a**, Cell lysates and SIGLEC12 immunoprecipitation samples from MEFs with CRISPR-induced knockout of SIGLEC12 were immunoblotted with the indicated antibodies. **b**, Cells were treated with TSE for 3 h, and cell death (CellToxGreen) or cell viability (CellTiterGlo) was quantified. **c**, Cells were transiently transfected with constitutively active mouse MLKL^Q343A^ or human MLKL^Q356A^ mutants for 24 h. Data were plotted as the mean ± s.d., representative of n = 9 (**b**, left and **c**) or n = 12 (**b**, right); three independent experiments. Statistical analyses were performed using two-tailed *t*-test. Data are representative of three independent experiments (**a**).

We did not find any inhibition of PMR during necroptosis in *Siglec12* knockout mouse cell lines (Extended Data Fig. 8a,b), suggesting that the pro-PMR activity of SIGLEC12 downstream of MLKL could be species-specific or possibly even human-specific. Notably, rodent amino acid sequences of SIGLEC12 orthologues substantially diverged from the primate sequences (Extended Data Fig. 3). Moreover, the human-specific *SIGLEC12* mutations that abolish its sialic-acid-binding property^12,14,15,23^ further reinforce the notion that the molecular role of SIGLEC12 in human cells could be unique to *Homo sapiens*. Consistently, the NISI motifs of human and mouse SIGLEC12 have sequence differences (Extended Data Fig. 3). Finally, constitutively active human MLKL^Q356A^ and its mouse counterpart MLKL^Q343A^ could both induce PMR in a SIGLEC12-independent manner in mouse cells (Extended Data Fig. 8c).

It is important to note that SIGLEC family conservation is convoluted and complex, and many species have completely different numbers of SIGLECs^24^. For example, the human genome has 16 family members, the macaque has 10, the marmoset has 6 and mice have 9. In mice, *Siglec12* is the closest orthologue of human *SIGLEC12* on the basis of sequence alignment^24^. The remaining eight mouse Siglecs are non-homologous to the human SIGLEC12, and none of the nine mouse orthologues has lost sialic-acid-binding capacity, unlike human SIGLEC12 (refs. ^12,25^). We could not find any literature or evidence for SIGLEC12 in Carnivora, which also do not have MLKL^26^. Thus, our results indicate that the human, but not mouse, plasma membrane requires SIGLEC12 to undergo PMR during necroptosis, revealing a potential uniqueness of human cells in this context.

Overall, these results indicate that SIGLEC12 is critical for PMR during necroptosis but dispensable for apoptosis, pyroptosis and ferroptosis, which are known to require NINJ1 for PMR; they thus suggest that the induction of PMR by SIGLEC12 downstream of MLKL is likely to be human-specific.

## SIGLEC12 is a membrane-rupturing molecule

**Fig. 4:**
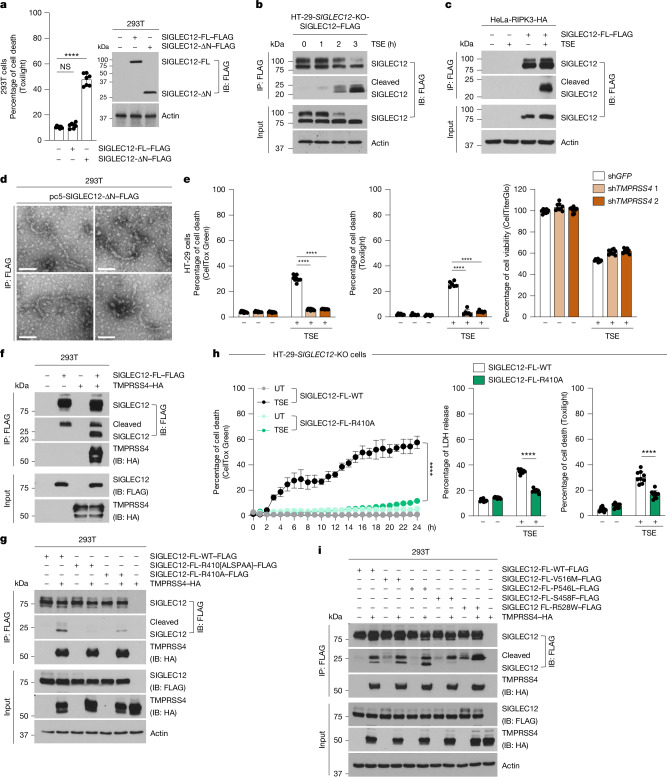
SIGLEC12 is cleaved by TMPRSS4 to induce PMR during necroptosis. **a**, 293T cells were transfected with full-length (SIGLEC12-FL–FLAG) or [22–410 amino acid]-truncated SIGLEC12 (SIGLEC12-ΔN–FLAG) for 40 h. Cell death was quantified using Toxilight (*P* = 0.183, *P *= 5.16 × 10^−7^), and protein expression levels were assessed using immunoblotting for FLAG. **b**,**c**, The HT-29-*SIGLEC12*-KO-SIGLEC12–FLAG stable cell line (**b**) or HeLa-RIPK3–HA cells (**c**) were treated with TSE for the indicated times (**b**) or for 4 h (**c**). Cell lysates and anti-FLAG immunoprecipitation samples were immunoblotted with the indicated antibodies. **d**, 293T cells were transiently transfected with SIGLEC12-ΔN–FLAG for 24 h, followed by anti-FLAG immunoprecipitation and analysis by transmission electron microscopy. **e**, HT-29 cells with the indicated knockdowns were treated with TSE for 8 h. Cell death or cell viability was quantified using the indicated assays (*P* = 2.77×10^−10^, *P *= 1.2 × 10^−9^, *P *= 6.59 × 10^−9^, *P *= 1.38×10^−7^). **f**,**g**,**i**, 293T cells were cotransfected with the indicated SIGLEC12-FL–FLAG plasmids with or without TMPRSS4–HA for 24 h. Cell lysates and anti-FLAG immunoprecipitation samples were immunoblotted with the indicated antibodies. The effect of TMPRSS4–HA expression on wild-type SIGLEC12 cleavage (**f**), cleavage of Arg cleavage site mutants (**g**), and cancer and general human population mutants (**i**) was detected. **h**, HT-29-*SIGLEC12*-KO-SIGLEC12-FL-WT or cleavage mutant (R410A) stable cell lines were treated with TSE, and cell death was assessed by green fluorescence intensity every hour for the indicated times using Incucyte S3 (*P *≤ 1 × 10^−15^, *P *= 4 × 10^−13^, *P *= 3.7 × 10^−6^). Data are plotted as the mean ± s.d., representative of *n* = 3 (**h**, left), *n* = 6 (**e**, middle), *n* = 7 (**a**) or *n* = 9 (**e**, left and right, **h**, middle and right). Statistical analyses were performed using two-tailed *t*-tests and two-way analyses of variance; ****P < 0.0001. Data are representative of three independent experiments (**a–c**, **f**, **g** and **i**). Scale bars, 100 nm (**d**).

**Extended Data Fig. 9:**
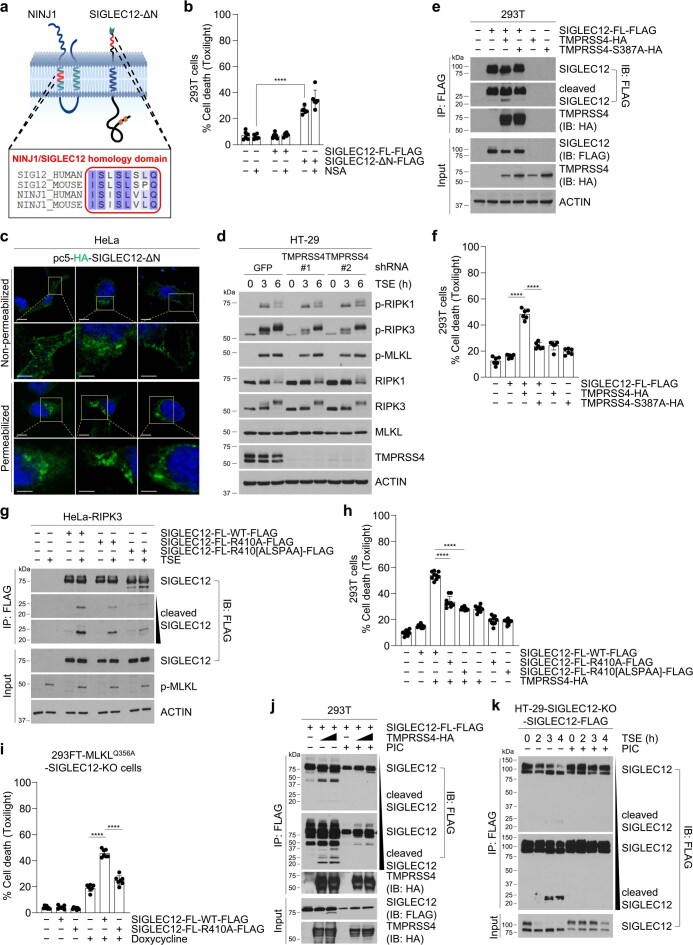
SIGLEC12 is cleaved by TMPRSS4 to induce plasma membrane rupture during necroptosis. **a**, Predicted structure of the [22-410aa]-truncated SIGLEC12 (SIGLEC12-ΔN). The red box shows the NINJ1/SIGLEC12 homology motif (NISI motif). **b**, 293 T cells were transfected with full-length (SIGLEC12-FL-FLAG) or [22-410aa]-truncated SIGLEC12 (SIGLEC12-ΔN-FLAG) for 40 h with or without NSA co-treatment. Cell death was measured using the Toxilight assay (p-value = 9.62 × 10^−8^). **c**, HeLa cells were transiently transfected with signal-peptide-HA-SIGLEC12-ΔN plasmid, and immunofluorescence was performed with or without cell permeabilization as indicated. Scale bars, 10 μm. **d**, HT-29 cells with shRNA-mediated stable knockdown of TMPRSS4 (two different shRNA sequences) were immunoblotted with the indicated antibodies following necroptosis induction by TSE. **e, f**, 293 T cells were co-transfected with indicated SIGLEC12-FLAG plasmids ± TMPRSS4-HA for 24 h. At 24 h post-transfection, cell lysates and anti-FLAG immunoprecipitation samples were immunoblotted with the indicated antibodies (**e**). Cell death was quantified at 40 h post-transfection by Toxilight assay (p-value = 9.69 × 10^−7^, 8.32 × 10^−7^) (**f**). **g**, HeLa-RIPK3-HA cells were transfected with indicated SIGLEC12-FLAG plasmids for 16 h and treated with TSE for 4 h. Cell lysates and anti-FLAG immunoprecipitation samples were analyzed by immunoblotting. **h, i**, 293 T cells were co-transfected with indicated SIGLEC12-FLAG plasmids ± TMPRSS4-HA for 40 h (**h**). 293FT-MLKL^Q356A^-SIGLEC12-KO cells were transfected with the indicated SIGLEC12-FLAG plasmids and treated with 0.1 μg/ml doxycycline for 24 h to induce MLKL-Q356A expression (**i**). Cell death was measured using the Toxilight assay (p-value = 9.74 × 10^−9^, 1.43 × 10^−10^ (**h**) and 7.79 × 10^−10^, 7.55 × 10^−8^ (**i**)). **j, k**, 293 T cells were co-transfected with SIGLEC12-FL-FLAG and TMPRSS4-HA for 24 h (**j**), and HT-29-SIGLEC12-KO-SIGLEC12-FLAG stable cell line was treated with TSE for 4 h with or without co-treatment with a protease inhibitor cocktail (PIC, cOmplete™, 1x final concentration) (**k**). Cell lysates and anti-FLAG immunoprecipitation samples were immunoblotted with the indicated antibodies. Data were plotted as the mean ± s.d., representative of n = 6 (**b**), n = 7 (**i**) or n = 9 (**f** and **h**); three independent experiments. Statistical analyses were performed using two-tailed *t*-test; ****p < 0.0001. Data are representative of three independent experiments (**d**, **e**, **g**, **j** and **k**). Panel **a** was created using Biorender (https://biorender.com).

To test the hypothesis that SIGLEC12 could directly induce PMR, we overexpressed full-length SIGLEC12 in 293T cells, but we did not detect significant cell death (Fig. 4a and Extended Data Fig. 9b). Following this, we proposed the hypothesis that SIGLEC12 activation during necroptosis could be required for its PMR-inducing capability to be unleashed. Indeed, we found that a small cleavage fragment of approximately 20 kDa was generated during necroptosis (Fig. 4b,c). This fragment was detected with an antibody against a carboxy-terminal FLAG tag, indicating that the extracellular region of SIGLEC12 was cleaved during necroptosis. Amino acid analysis of the region for trypsin-like protease sites revealed a conserved Arg410 (which was notably not conserved in rodents) that fit this motif, with cleavage at this residue predicted to yield the observed 20-kDa fragment; notably, this fragment contained the NISI motif (Extended Data Figs. 3 and 9a).

We generated a truncation mutant (SIGLEC12-ΔN) that lacked the amino acids between the signal peptide and Arg410, which would mimic SIGLEC12 cleavage at that residue by a hypothetical trypsin-like extracellular or transmembrane protease (Extended Data Fig. 9a). Notably, expression of SIGLEC12-ΔN but not the full-length SIGLEC12 (SIGLEC12-FL) resulted in significant PMR induction (Fig. 4a). This PMR was not blocked by NSA, suggesting that SIGLEC12-ΔN-induced PMR is not MLKL-dependent; this was consistent with SIGLEC12 cleavage during necroptosis downstream of MLKL being a key step in the activation of its ability to induce PMR (Extended Data Fig. 9b). Finally, expression of the cleavage-mimetic SIGLEC12-ΔN was sufficient to form both cytosolic and membrane-associated puncta (Extended Data Fig. 9c) and produce fibrils (Fig. 4d), suggesting that the cleavage event is a critical and probably sufficient step in the activation of SIGLEC12 that drives fibril assembly.

## SIGLEC12 is activated by TMPRSS4 during necroptosis

To determine the mechanism of SIGLEC12 cleavage during necroptosis, we mined the NCBI Gene database for reported interactors with SIGLEC12 that possess protease activity. Notably, an interaction with a transmembrane protease called TMPRSS4 has previously been reported in a yeast two-hybrid screen^27^. TMPRSS4 is a type II serine protease that has been linked to viral infections and cancer^28–31^. Its role in necroptosis is not known. We proposed that TMPRSS4 could be responsible for cleavage of the extracellular region of SIGLEC12, leading to its activation during necroptosis. Indeed, *TMPRSS4* knockdown inhibited PMR during necroptosis without altering the upstream signalling (Fig. 4e and Extended Data Fig. 9d); in addition, TMPRSS4 physically interacted with SIGLEC12 (Fig. 4f,g), and coexpression of the wild-type but not the protease-dead S387A mutant of TMPRSS4 with SIGLEC12–FLAG induced SIGLEC12 cleavage and PMR (Fig. 4f and Extended Data Fig. 9e,f). Finally, TMPRSS4-induced SIGLEC12 cleavage and PMR were inhibited by mutation of the R410 residue (Fig. 4g,h and Extended Data Fig. 9g–i) and by supplementation of the cell culture medium with a protease inhibitor cocktail (Extended Data Fig. 9j,k).

In summary, these results demonstrate that TMPRSS4 is key for extracellular cleavage and activation of SIGLEC12 and consequent PMR during necroptosis, as it converts SIGLEC12 from its precursor form to its active form, allowing induction of PMR by SIGLEC12.

## SIGLEC12 mutations in cancer and the human population

Loss of necroptosis potential is frequently found in cancer cells^32^. According to The Cancer Genome Atlas (TCGA), 444 missense and 63 truncating mutations have been reported in the exons of *SIGLEC12* in 67,030 individuals with cancer (somatic mutation frequency of 0.7%). We found that the top missense *SIGLEC12* mutation reported in TCGA (Ser458Phe) inhibited the ability of SIGLEC12 to be cleaved by TMPRSS4 (Fig. 4i). Notably, the Ser458Phe mutation is located in the NISI motif of SIGLEC12, suggesting that this motif may have a role in the cleavage and activation of SIGLEC12 (Extended Data Figs. 3 and 9a). These findings indicate that inactivation of the pro-PMR role of SIGLEC12 may contribute to resistance to necroptotic cell death in cancer.

**Extended Data Fig. 10:**
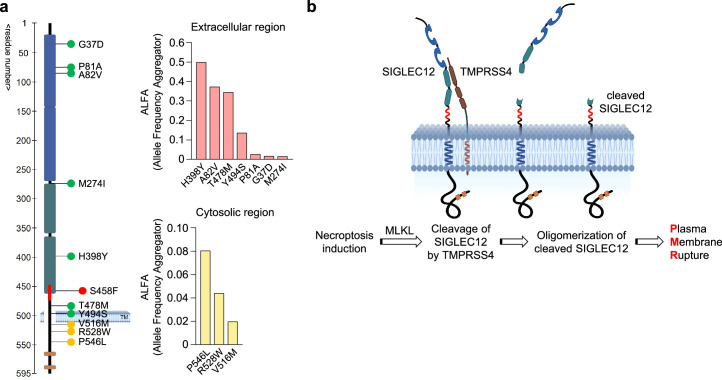
SIGLEC12 mutations and the proposed model of TMPRSS4-mediated SIGLEC12 activation during necroptosis. **a**, Summary of the SIGLEC12 mutation frequencies found in the general population (graphs) and their location in the SIGLEC12 domains. Green circles: extracellular mutations; Red circle: cancer-associated mutation S458F; Yellow circles: intracellular mutations; TM: transmembrane region. Blue boxes: V-type Ig domains; Green boxes: C2-type Ig domains; Orange boxes: ITIM and ITSM domains; Red box: NISI motif. **b**, A proposed model of SIGLEC12 activation and plasma membrane rupture induction during necroptosis. Panels **a** and **b** were created using Biorender (https://biorender.com).

Functional human *MLKL* mutations that are present in 2–3% of the world population have been previously reported^33^. We mined the NCBI dbSNP database^34,35^ for SIGLEC12 variants found in the normal human population and found three top variants in the cytosolic region of the protein: Val516Met, Arg528Trp and Pro546Leu (Extended Data Fig. 10a). Notably, similar to the cancer-associated Ser458Phe variant, the Arg528Trp mutation blocked SIGLEC12 cleavage by TMPRSS4 (Fig. 4i). The ramifications of these SIGLEC12 sequence variations on human physiology remain to be elucidated.

Furthermore, an insG mutation has been suggested as a frameshift mutation (Ala66fs) in exon 1 of *SIGLEC12* that is present in the general population at a substantial frequency—ranging from 38% to 86% (depending on the region)—and at various zygosities^14^. As this insertion mutation is in exon 1 of *SIGLEC12*, it remains to be determined whether this exon is spliced out in a cell-specific or tissue-specific manner, or whether *SIGLEC12* expression occurs through multiple transcriptional start sites, allowing the rest of the open reading frame of *SIGLEC12* to be expressed, especially when such a mutation is present. Such alternative transcription or splicing could also be triggered during necroptosis-activating conditions, such as infection, allowing expression of *SIGLEC12* that misses the exon 1 residues (1–142), which we have shown are part of the SIGLEC12 region that is cleaved away during necroptosis. However, an isoform missing exon 1 would require a cryptic signal peptide, as exon 1 contains the signal peptide. Notably, a *SIGLEC12* isoform missing exon 1 has been reported^36^. However, given the high frequency of the frameshift mutation, it is conceivable that defects in PMR because of this mutation could provide selective fitness owing to the inability of the infected cells to release the pathogens that rely on cell lysis for propagation.

Our data show that MLKL and SIGLEC12 interact before SIGLEC12 is cleaved and that inhibition of MLKL by NSA attenuates SIGLEC12 cleavage (Fig. 3h and Extended Data Fig. 6f). These findings point to a model in which the role of MLKL upstream of SIGLEC12 is to promote cleavage of SIGLEC12 by TMPRSS4. Future studies will identify the molecular mechanisms that enable SIGLEC12 cleavage by TMPRSS4 downstream of MLKL activation during necroptosis.

The residues in the NISI motif of NINJ1 (I^84^SISLVLQ^91^ in human NINJ1) have been shown to be critical for its PMR function: I84F and Q91A mutations reduced its ability to homo-oligomerize and mediate PMR, whereas I86F and L90W reduced its ability to mediate PMR^20,37,38^. These findings further highlight the functional importance of this motif. Whether SIGLEC12 is the sole mediator of PMR during necroptosis or whether NINJ1 also contributes to this process, possibly in a tissue-specific, cell-type-specific and/or necroptosis-inducing trigger-specific manner, remains to be determined. It is worth noting that a recent study showed that MLKL could target mitochondrial membranes^39^; this could partially or fully explain the loss of ATP levels during necroptosis without loss of plasma membrane integrity in the absence of SIGLEC12 (Fig. 2a,d), as mitochondrial membrane permeabilization would significantly affect cellular ATP production.

Overall, our work identifies SIGLEC12 as a protein that is critical for PMR during necroptosis, with feature and sequence similarities to NINJ1 (Extended Data Fig. 10b). Whether cancer-associated mutations in *SIGLEC12* or its variants found in the general population play an important part in the pathogenesis of human diseases, such as sensitivity to infections, in which necroptosis has been implicated, remains to be determined. Our discovery of SIGLEC12 as a species-specific and possibly human-specific mediator of PMR during necroptosis sheds light on the complexity of how programmed necrosis is executed and offers both SIGLEC12 and TMPRSS4 as druggable targets for modulating proinflammatory cell death in human diseases.

## Methods

### Antibodies and reagents

The following antibodies were used in this study: RIPK1 (Cell Signaling Technology, catalogue no. 3493); p-hRIPK1 (Cell Signaling Technology, catalogue no. 65746); hRIPK3 (Cell Signaling Technology, catalogue no.13526); p-hRIPK3 (Cell Signaling Technology, catalogue no. 93654); hMLKL (Cell Signaling Technology, catalogue no. 14993; Abcam, ab183770; Thermo Fisher Scientific, PA5-34733); p-hMLKL (Cell Signaling Technology, catalogue no. 91689); anti-SIGLEC12 (Boster, A10550-1; Invitrogen, PA5-110369; Invitrogen, PA5-31457); TMPRSS4 (Cell Signaling Technology, catalogue no. 84382); anti-HMGB1 (Cell Signaling Technology, catalogue no. 6893); anti-β-actin (Sigma-Aldrich, A5316); anti-FLAG (Sigma-Aldrich, F1804); anti-FLAG–HRP (Cell Signaling Technology, catalogue no. 86861); anti-mouse IgG (Cell Signaling Technology, catalogue no. 7074); anti-rabbit IgG (Cell Signaling Technology, catalogue no. 7076), anti-mouse IgG (H + L) and F(ab′)2 Fragment (Alexa Fluor 488 Conjugate) (Cell Signaling Technology, catalogue no. 4408). All primary antibodies were used at 1:2,000 dilution. All secondary antibodies were used at 1:5,000 dilution.

The following reagents were used in this study: SM-164 (S7089), Nec-1s (S8641), GSK′872 (S8465), NSA (S8251), emricasan (S7775) and erastin (S7242) from SelleckChem; etoposide (E1383), α-ketoglutarate (349631), lipopolysaccharide (L4391), luminol (A8511), *p*-coumaric acid (C9008) and anti-FLAG M2 agarose beads (M8823) from Sigma-Aldrich; nigericin (11437) from the Cayman Chemical Company; Lipofectamine 3000 (L3000015), SuperSignal West Atto Ultimate Sensitivity Substrate (A38556) and Prolong Diamond Antifade Mountant with DAPI (P36966) from Thermo Fisher Scientific; polyethylenimine (PEI; 24765-100) from Kyfora Bio; polybrene (TR-1003) from EMD Millipore; and 10X Tris/Glycine/SDS Electrophoresis Buffer (1610772), Tween 20 (1610781), Stacking Gel Buffer for PAGE (1610799), Resolving Gel Buffer for PAGE (1610798), Precision Plus Protein Dual Color Standards (1610394) and nitrocellulose membrane (1620115) from Bio-Rad. Autoradiography films were from MTC Bio (A8815).

### Cell lines and growth media

The 293T, HEK293-FlpIn-TREx, HeLa and MEF cells were grown in DMEM (Cytiva, SH30243.FS), with l-glutamine, 4.5 g l^−1^ glucose and pyruvate; HT-29 cells were grown in McCoy’s 5A medium (Gibco, 16600-082, with l-glutamine); and A549, Jurkat and THP-1 cells were grown in RPMI 1640 medium (Fisher Scientific, SH30255.01, with HEPES and l-glutamine). All media were supplemented with 10% fetal bovine serum (GeminiBio), 1× non-essential amino acids (Cytiva, SH30238.01) and 1× antibiotic/antimycotic solution (Sigma-Aldrich, A5955). All cell lines were regularly tested for mycoplasma contamination using Lonza’s MycoAlert Kit (catalogue no. LT07-318) and tested negative for mycoplasma.

### Human tumour and normal tissue samples

Deidentified human benign (normal) lung and colon and lung and colon adenocarcinoma tumour specimens were obtained from the University of Texas Southwestern Tissue Management Shared Resource. Patients were enrolled and consented to a protocol approved by the institutional review board. Formalin-fixed tissues were processed as in the ‘Immunohistochemistry of tissue slices’ section.

### Molecular cloning and plasmids

Molecular cloning was performed using New England Biolabs restriction enzymes and T4 DNA ligase. Plasmids were transformed into chemically competent NEB Stable (for lentiviral plasmids, at 30 °C) and DH5α *Escherichia coli* cells (for non-lentiviral plasmids at 37 °C). Plasmid purification and extraction were performed using a QIAprep Spin Miniprep Kit (Qiagen, 27106) and QIAquick Gel Extraction Kit (Qiagen, 28704). sgRNAs targeting SIGLEC12 were cloned into pSpCas9(BB)-2A-GFP (pX458) (Addgene, catalogue no. 48138). Cloned plasmids were amplified and purified using a ZymoPURE II Plasmid Midiprep Kit (Zymo Research, D4201).

### Generation of knockout cell lines using CRISPR–Cas9

Cells were transfected with the pSpCas9(BB)-2A-GFP (pX458) plasmids for 24 h using Lipofectamine 3000. Cells expressing the highest levels of GFP were then sorted into 96-well plates containing 150 µl of high-glucose DMEM or McCoy’s medium supplemented with 16% fetal bovine serum, 1× non-essential amino acids and 1× antibiotic/antimycotic. All flow cytometry experiments were performed at the UT Southwestern Cell Sorting Facility. Fluorescence-activated cell sorting was performed with a BD FACSAria II flow cytometer under sterile conditions using a 100 nozzle at 37 °C. About 2–3 weeks later, the clones were expanded into 12-well plates. Cells were lysed in 2× SSB (150 mM Tris-HCl, pH 6.8, 4% SDS, 0.01% bromophenol blue, 20% glycerol) + 2% β-mercaptoethanol (β-ME), and lysates were analysed for knockout screening using immunoblotting.

### Generation of knockdown cell lines using lentiviral short hairpin RNA

MISSION short hairpin RNA plasmids targeting *SIGLEC12*, *TMPRSS4* and *MLKL* were from Sigma-Aldrich. For generation of lentiviral particles, plasmids were transfected into 293T cells using PEI (3 μl per 1 μg DNA). Pseudoviral particles were collected 72 h after transfection, and cells were transduced in the presence of polybrene (8 μg ml^−1^). Cells were selected with puromycin (2 μg ml^−1^) for 48 h after transduction, and knockdown of target proteins was confirmed by immunoblotting after all cells in the untransduced control plates had been selected out.

### Transfection experiments

Cells were transfected with a total of 1 µg of plasmid DNA or 3 µg of plasmid DNA in a 24-well plate or 100 mm dish, respectively. The ratio of pcDNA5-SIGLEC12–FLAG to pLenti-III-EF1-TMPRSS4–HA plasmid DNA was 1:1, and the total amount was adjusted using an empty vector. HeLa-RIPK3–HA cells were transfected using Lipofectamine 3000 (1.5 μl per 1 μg plasmid DNA), whereas 293T cells were transfected using PEI (3 μl per 1 μg plasmid DNA).

### Cell death and viability assays

Necroptosis was induced with TSE cocktail (TSE; T: 30 ng ml^−1^ hTNF, S: 0.2 µM SM-164, E: 5 µM emricasan; 1 h pretreatment for S + E) for 8 h. Extrinsic apoptosis was induced with T and S (1 h pretreatment with S). Intrinsic apoptosis was induced with 100 µM etoposide. Pyroptosis was induced with α-ketoglutarate (15 mM) or LPS (1 μg ml^−1^, 4 h pretreatment) + nigericin (20 μM). Ferroptosis was induced with erastin (20 μM). CellToxGreen (Promega, G8731), ToxiLight Non-destructive Cytotoxicity BioAssay (Lonza, LT07-117) and CytoTox 96 Non-Radioactive Cytotoxicity Assay (LDH assay, Promega, G1780) were used for detection of cell death. Cell viability was measured using a CellTiter-Glo Luminescent Cell Viability Assay (Promega, G7570). For CellToxGreen, dye (used at 1:3,000 dilution) was added to the wells immediately before the fluorescence reads. For the Toxilight assay, 12.5 µl of culture medium was collected in triplicate and mixed with an equal volume of Toxilight reagent in 384-well plates. LDH assay was performed according to the manufacturer’s instructions, and absorbance was measured at 490 nm. For the cell survival assay, CellTiter-Glo reagent was added directly to the medium. Cells were lysed for 10 min in the dark at 25 °C, and luminescence was measured. Cell survival was normalized to that of an untreated control.

### Live-cell imaging of cell death using Incucyte S3

Cells were seeded into 24-well plates and treated the following day. Plates were imaged using an Incucyte S3 Live-Cell Analysis System (Sartorius) with scans every 1 h using a ×10 objective, capturing phase contrast under the AI Scan module. Quantification of cell death was performed using the integrated AI Cell Health Analysis module, which applies a deep learning model to distinguish living and dead cells on the basis of morphology. Cell death was expressed as the percentage of dead cells per total cell count of cumulative cell counts over time.

### Sample preparation for immunoblotting

Cells were lysed in 200 µl of 2× SSB + β-ME buffer. The plates were heated at 90 °C for 3 min, then cooled to room temperature for 5 min, and 1–2 µl of 1× benzonase (1:10 diluted with 50% glycerol from 10× supplier stock to obtain 1×; Santa Cruz Biotechnology, sc-202391) was added to the lysates to degrade genomic DNA for 5 min at room temperature, on a rocker. Total protein levels were normalized using a BCA kit (Thermo Fisher Scientific, 23225) or reducing-agent-compatible BCA kit (Thermo Fisher Scientific, 23250). For non-reducing sample preparation, cells were lysed in NLB buffer (NP-40 lysis buffer: 25 mM HEPES (pH 7.5), 0.2% NP-40, 120 mM NaCl, 0.27 M sucrose, 5 mM EDTA, 5 mM EGTA, 50 mM NaF, 10 mM *b*-glycerophosphate, 5 mM sodium pyrophosphate, 1 mM Na3VO4 (fresh), 0.1% β-ME, 1 mM phenylmethylsulfonyl fluoride (fresh), 2× complete protease inhibitor cocktail (Roche, 80024400)). Cell lysates were mixed with 4× SDS sample buffer without β-ME (non-reducing). Equal amounts of protein were resolved by SDS–PAGE and analysed using the indicated antibodies.

### Immunoblotting

Total cell lysates or pull-down samples were heated at 90 °C for 5 min or 10 min in 2× or 4× SSB buffer, respectively, then subjected to 10% or 15% SDS–PAGE. Proteins were electrotransferred on to nitrocellulose membranes for 1.5 h at 0.4 A with the wet transfer tank submerged in an ice bath. The membranes were blocked for 1 h in TBST buffer containing 5% (w/v) skimmed milk and then incubated with the primary antibodies in TBST containing 5% (w/v) bovine serum albumin + 0.05% NaN_3_ overnight at 4 °C. Detection was carried out using HRP-conjugated secondary antibodies and a homemade chemiluminescence reagent (2.5 mM luminol, 0.4 mM *p*-coumaric acid, 100 mM Tris-HCl, pH 8.6, 0.018% H_2_O_2_).

### Immunoprecipitation

Cells were seeded into a 100-mm dish at 50% confluence. After 24 h, cells were transfected with total 3 µg of plasmid DNA using 9 μl of PEI or 4.5 µl of lipofectamine 3000. After 14–24 h, cells were lysed in NRP buffer. Cell lysates were incubated and precipitated with specific primary antibody for overnight at 4 °C and then incubated with protein A/G-magnetic beads (Thermo Fisher Scientific, 88802) or anti-FLAG agarose beads (Sigma-Aldrich, M8823) for 4 h at 4 °C. Bound proteins were removed by boiling in 2× SSB + β-ME buffer for 10 min at 90 °C, separated by SDS–PAGE and immunoblotting, and visualized using a homemade chemiluminescence reagent.

### Time-lapse live-cell imaging microscopy

Cells were seeded on a 24-well plate (Cellvis, P24-1.5 P). After 48 h, cells were treated with TSE with added CellToxGreen. Live-cell imaging was performed using a Nikon Spinning Disk Confocal CSU-W1 with a ×40 air objective. Both bright-field and GFP fluorescence channels were captured every 30 min for 24 h. During the live-cell imaging process, cells were maintained at 37 °C and 5% CO_2_.

### Immunofluorescence microscopy

Cells were seeded on 70% ethanol-sterilized glass coverslips (AmScope, CS-R18-100). After 24 h, cells were transfected with total 0.5 µg of plasmid DNA using 0.75 µl of lipofectamine 3000. Cells were treated with TSE and then fixed in 4% paraformaldehyde for 12 min. Cells were washed twice with phosphate-buffered saline (PBS), with or without permeabilization using 0.05% Triton X-100 for 5 min. After incubation in a blocking buffer (1% bovine serum albumin in PBS) for 1 h, the cells were incubated overnight at 4 °C with the following primary antibodies: anti-FLAG or anti-HA. They were then incubated with the following Alexa Fluor secondary antibodies for 1 h at room temperature. Cells were mounted using Prolong Diamond Antifade Mountant with DAPI. Images were obtained with Laser scanning confocal Zeiss LSM880 inv. + Airyscan confocal, magnification 63x. Images were analysed using Fiji/ImageJ. Images are representative of at least ten fields of view per each sample.

### Immunohistochemistry of tissue slices

Immunohistochemical analyses were conducted using a Dako Autostainer Link 48 system. Initially, the slides were baked at 60 °C for 20 min, followed by deparaffinization and hydration. Heat-induced antigen retrieval was performed using the Dako PT Link. The tissue samples were treated with a peroxidase block, and antibody incubations were carried out at a 1:200 dilution. Staining was visualized with a Nikon Widefield Epi-scope, magnification ×60. For Extended Data Fig. 4c, images were quantified using Fiji. In brief, the total tissue area was manually outlined, not considering large blood vessels. Then, the raw data was colour-deconvolved using the Colour Deconvolution2 plugin and thresholded manually to include the darkest SIGLEC12 staining. Results were expressed as the percentage area of SIGLEC12 relative to total tissue area.

### Cell plug preparation

Cells were seeded in 100-mm culture dishes at approximately 80% confluence. After 24 h, cells were rinsed with 10 ml of cold PBS and fixed in 10 ml of 4% paraformaldehyde for 20 min at room temperature in the dark. Cells were then washed again with cold PBS and gently scraped into 1 ml of PBS. The suspension was centrifuged at 300*g* for 5 min at room temperature, and the pellet was resuspended in 100 μl of PBS and kept on ice. Separately, 20 ml of 1% (w/v) agarose in PBS was prepared by boiling and then cooled. A 500-μl aliquot of molten agarose was transferred to a sterile 2-ml microcentrifuge tube, and 100 μl of the cell suspension was added. The mixture was gently inverted 20 times to ensure even distribution, then centrifuged at 300*g* for 5 min at room temperature. The resulting cell plugs were maintained on ice for 1 h to solidify and stored at 4 °C for up to 2 days before processing for sectioning, haematoxylin and eosin staining, and preparation of unstained slides.

### Electron microscopy

For transmission electron microscopy, carbon grids with a 400-mesh size (Electron Microscopy Sciences, CF-400-Cu-50) were subjected to glow discharge using a PELCO easiGlow Discharge Cleaning System for 25 s. Then, 5 µl of the purified fibril sample was applied to the grid and left to incubate for 1 min before being removed with filter paper. The grid was then stained with 5 µl of filtered aqueous 2% uranyl acetate solution (Electron Microscopy Sciences, catalogue no. 22400) for 1 min; excess stain was absorbed using filter paper. After drying, the grid was imaged using a JEM-1400 Plus transmission electron microscope, equipped with a LaB6 source operating at 120 kV and an AMT-BioSprint 16M CCD camera. For scanning electron microscopy, samples were fixed with 2.5% (v/v) glutaraldehyde in 0.1 M sodium cacodylate buffer overnight at 4 °C. After three rinses in 0.1 M sodium cacodylate buffer, they were postfixed with 2% osmium tetroxide in 0.1 M sodium cacodylate buffer for 2 h. The samples were then rinsed with water and dehydrated with increasing concentrations of ethanol, followed by increasing concentrations of hexamethyldisilazane in ethanol. Cells on coverslips were air-dried under a hood, mounted on scanning electron microscopy stubs with carbon tape, and sputter-coated with gold/palladium using a Cressington 108 auto sputter coater. Images were acquired with a field-emission scanning electron microscope (Zeiss Sigma) at an accelerating voltage of 3 kV and a 15-degree tilt.

### Genome-wide CRISPR-based knockout screens

Human Brunello sgRNA library (Addgene, catalogue no. 73178-LV) was used^40^. This library contains 76,441 sgRNAs targeting 19,114 genes (4 sgRNAs per gene), with 1,000 non-targeting controls^40^. HEK293-FlpIn-TREx-MLKL^Q356A^ cells were transduced at a multiplicity of infection of 0.5 using 8 µg ml^−1^ polybrene, and the plates were spun at 1,000*g* for 60 min at room temperature (spinfection). Two days later, cells were expanded and selected with 2 µg ml^−1^ puromycin for 2 weeks. The knockout library was treated with 0.1 µg ml^−1^ doxycycline to induce MLKL^Q356A^ expression and necroptotic cell death for 3 days (with daily feeding for 3 days to remove dead cells). Genomic DNA from the surviving cells was isolated (using Tissue/Blood DNeasy kit from Qiagen; catalogue no. 69504), and PCR was performed using Emerald Taq (Takara Bio, catalogue no. RR310B) with P7 and P5 primers to amplify the sgRNA sequences, which were then subjected to next-generation sequencing on an Illumina NextSeq 500 with a read configuration of 100 bp, single-end. All the fastq files underwent routine quality checks using FastQC (v.0.11.2; http://www.bioinformatics.babraham.ac.uk/projects/fastqc) and FastQ Screen (v.0.4.4; http://www.bioinformatics.babraham.ac.uk/projects/fastq_screen). The trimmed fastq files were mapped to the reference sgRNA library with a mismatch option set to 0 using MAGeCK. Read counts for each sgRNA were generated, and median normalization was performed to adjust for library sizes. Positively and negatively selected sgRNAs and genes were identified using the default parameters of MAGeCK.

### Quantitative real-time PCR

Total RNA was extracted using TRIzol reagent (Thermo Fisher Scientific) according to the manufacturer’s instructions. Cells were lysed directly in TRIzol, and RNA was purified by chloroform phase separation and isopropanol precipitation. The RNA pellet was washed with 75% ethanol, air-dried and resuspended in RNase-free water. RNA concentration and purity were determined using a NanoDrop spectrophotometer (Thermo Fisher Scientific). cDNA was synthesized from 1 μg total RNA using EcoDry Premix with Random Hexamers (Takara Bio, catalogue no. 639547). The RNA was added directly to the lyophilized premix, incubated at 42 °C for 1 h and heat-inactivated at 85 °C for 5 min. Quantitative PCR was performed using SYBR Green PCR Master Mix (Applied Biosystems) on a QuantStudio 5 Real-Time PCR System (Thermo Fisher Scientific). Each reaction was run in triplicate with the following cycling conditions: 95 °C for 2 min, followed by 40 cycles of 95 °C for 10 s, 60 °C for 30 s and 72 °C for 30 s. Gene expression was normalized to that of *ACTB* using the ΔΔCt method, and data were expressed as fold change relative to control samples. Expression of inflammatory cytokines and chemokines was assessed using gene-specific primers as follows: *CXCL1* (forward 5′-AGGGAATTCACCCCAAGAAC-3′, reverse 5′-TGGATTTGTCACTGTTCAGCA-3′); *TNFA* (forward 5′-CAGAGGGCCTGTACCTCATC-3′, reverse 5′-GGAAGACCCCTCCCAGATAG-3′); *IL1B* (forward 5′-AAGTACCTGAGCTCGCCAGTGA-3′, reverse 5′-TGCTGTAGTGGTGGTCGGAGAT-3′); *CXCL8* (forward 5′-TCTGCAGCTCTGTGTGAAGG-3′, reverse 5′-AATTTCTGTGTTGGCGCAGT-3′).

### Proteomics

Samples were digested overnight with trypsin (Pierce) following reduction and alkylation with DTT and iodoacetamide (Sigma-Aldrich). Following solid-phase extraction cleanup with an Oasis HLB µElution Plate (Waters), the resulting peptides were reconstituted in 10 µl of 2% (v/v) acetonitrile (ACN) and 0.1% trifluoroacetic acid in water. Then, 2 µl of each sample was injected into an Orbitrap Fusion Lumos mass spectrometer (Thermo Electron) coupled to an Ultimate 3000 RSLC-Nano liquid chromatography system (Dionex). Samples were injected into a 75 μm i.d., 75-cm-long EasySpray column (Thermo) and eluted with a gradient from 0–28% buffer B over 90 min. Buffer A contained 2% (v/v) ACN and 0.1% formic acid in water, and buffer B contained 80% (v/v) ACN, 10% (v/v) trifluoroethanol and 0.1% formic acid in water. The mass spectrometer was operated in positive ion mode with a source voltage of 1.5 kV and an ion transfer tube temperature of 300 °C. Mass spectrometry scans were acquired at 120,000 resolution in the Orbitrap, and up to ten tandem mass spectra spectra were obtained in the Orbitrap for each full spectrum acquired using higher-energy collisional dissociation for ions with charges 2–7. Dynamic exclusion was set for 25 s after an ion had been selected for fragmentation. Raw mass spectrometry data files were analysed using Proteome Discoverer v.2.4 SP1 (Thermo), with peptide identification performed using Sequest HT searching against the human reviewed protein database from UniProt^41^. Fragment and precursor tolerances of 10 ppm and 0.6 Da were specified, and three missed cleavages were allowed. Carbamidomethylation of Cys was set as a fixed modification, and oxidation of Met was set as a variable modification. The false discovery rate cutoff was 1% for all peptides.

### Statistical and bioinformatics analysis

For all experiments, unless otherwise indicated, *n* was at least 3. Statistical analyses were performed using Prism (GraphPad Software). Data were analysed using one-way analysis of variance with Bonferroni post-test. Student’s *t*-test was used for paired datasets. Data points indicate the mean ± s.d. Alignments were done using Clustal Omega^42^ and visualized using Jalview^43^. Secondary structure predictions were obtained from UniProt^41^.

### ProteinAtlas data analysis

The clustering data for the gene expression sets from the deep sequencing of RNA from 40 different normal tissue types (https://www.proteinatlas.org/ENSG00000254521-SIGLEC12/tissue) and scRNA-seq clustering data from 31 human tissue types (https://www.proteinatlas.org/ENSG00000254521-SIGLEC12/single+cell) were obtained from the ProteinAtlas database (v.22)^44^. UMAP plots display gene clusters from Louvain clustering of gene expression across all tissue types or single cell types.

### Human mutation analyses

To identify the most prevalent *SIGLEC12* point mutations in the general population, we conducted a comprehensive analysis using the dbSNP database. We specifically targeted missense mutations owing to their potential impact on protein function. The search involved filtering for missense mutations in the specified genes and extracting the relevant data. We isolated the necessary columns and ranked the mutations in descending order on the basis of allele frequency aggregator (ALFA) values. The ALFA value represents the aggregation of allele frequency across diverse populations, providing a comprehensive measure of mutation prevalence. From this analysis, we identified the top ten mutations with the highest ALFA values, implicating the most significant missense mutations in the general population. To identify the most frequent *SIGLEC12* mutations associated with cancer, TCGA was analysed using cBioportal^45^.

### Reporting summary

Further information on research design is available in the Nature Portfolio Reporting Summary linked to this article.

## Online content

Any methods, additional references, Nature Portfolio reporting summaries, source data, extended data, supplementary information, acknowledgements, peer review information; details of author contributions and competing interests; and statements of data and code availability are available at 10.1038/s41586-025-09741-1.

## Supplementary information

Supplementary Fig. 1

Reporting Summary

Supplementary Video 1 Time-lapse live-cell imaging confocal microscopy of PMR in HT-29-shGFP stable cell line during necroptosis (bright-field and CellToxGreen channels). Stills are shown in Fig. 2d (left panel). HT-29-shGFP stable shRNA-mediated knockdown cell line was treated with TSE for 24 h, and cell death was assessed using CellToxGreen and time-lapse fluorescence confocal microscopy. A merge of bright-field and green fluorescence channels is shown. Representative of three independent experiments.

Supplementary Video 2 Time-lapse live-cell imaging confocal microscopy of PMR in HT-29-shGFP stable cell line during necroptosis (CellToxGreen channel). Stills are shown in Fig. 2d (left panel). HT-29-shGFP stable shRNA-mediated knockdown cell line was treated with TSE for 24 h, and cell death was assessed using CellToxGreen and time-lapse fluorescence confocal microscopy. The green fluorescence channel is shown. Representative of three independent experiments.

Supplementary Video 3 Time-lapse live-cell imaging confocal microscopy of PMR in HT-29-shSIGLEC12 stable cell line during necroptosis (bright-field and CellToxGreen channels). Stills are shown in Fig 2d (left panel). HT-29-shSIGLEC12 stable shRNA-mediated knockdown cell line was treated with TSE for 24 h, and cell death was assessed using CellToxGreen and time-lapse fluorescence confocal microscopy. A merge of bright-field and green fluorescence channels is shown. Representative of three independent experiments.

Supplementary Video 4 Time-lapse live-cell imaging confocal microscopy of PMR in HT-29- shSIGLEC12 stable cell line during necroptosis (CellToxGreen channel). Stills are shown in Fig. 2d (left panel). HT-29-shSIGLEC12 stable shRNA-mediated knockdown cell line was treated with TSE for 24 h, and cell death was assessed using CellToxGreen and time-lapse fluorescence confocal microscopy. The green fluorescence channel is shown. Representative of three independent experiments.

Supplementary Video 5 Time-lapse live-cell imaging confocal microscopy of PMR in HT-29-shGFP stable cell line during necroptosis (high magnification of the bright-field channel). Stills are shown in Fig. 2d (right panel). HT-29-shGFP stable shRNA-mediated knockdown cell line was treated with TSE for 24 h, and time-lapse confocal microscopy was performed. The bright-field channel is shown. Representative of three independent experiments.

Supplementary Video 6 Time-lapse live-cell imaging confocal microscopy of PMR in HT-29-shSIGLEC12 stable cell line during necroptosis (high magnification of the bright-field channel). Stills are shown in Fig. 2d (right panel). HT-29-shSIGLEC12 stable shRNA-mediated knockdown cell line was treated with TSE for 24 h, and time-lapse confocal microscopy was performed. The bright-field channel is shown. Representative of three independent experiments

## Data Availability

All data reported in this paper are available on request. Data from the NCBI dbSNP database (https://www.ncbi.nlm.nih.gov/snp/?term=SIGLEC12) and ProteinAtlas database (https://www.proteinatlas.org/ENSG00000254521-SIGLEC12/tissue; https://www.proteinatlas.org/ENSG00000254521-SIGLEC12/single+cell) were used in this study.

## Code availability

All code reported in this manuscript are available on request.
