# Supplementary material for: SIGLEC12 mediates plasma membrane rupture during necroptotic cell death
Source: Nature. 2025 Nov 12;649(8096):460–6. doi: 10.1038/s41586-025-09741-1 (PMC12779560; doi:10.1038/s41586-025-09741-1)

nature portfolio

https://doi.org/10.1038/s41586-025-09741-1

Supplementary information

SIGLEC12 mediates plasma membrane rupture during necroptotic cell death

In the format provided by the authors and unedited

Fig. 2.

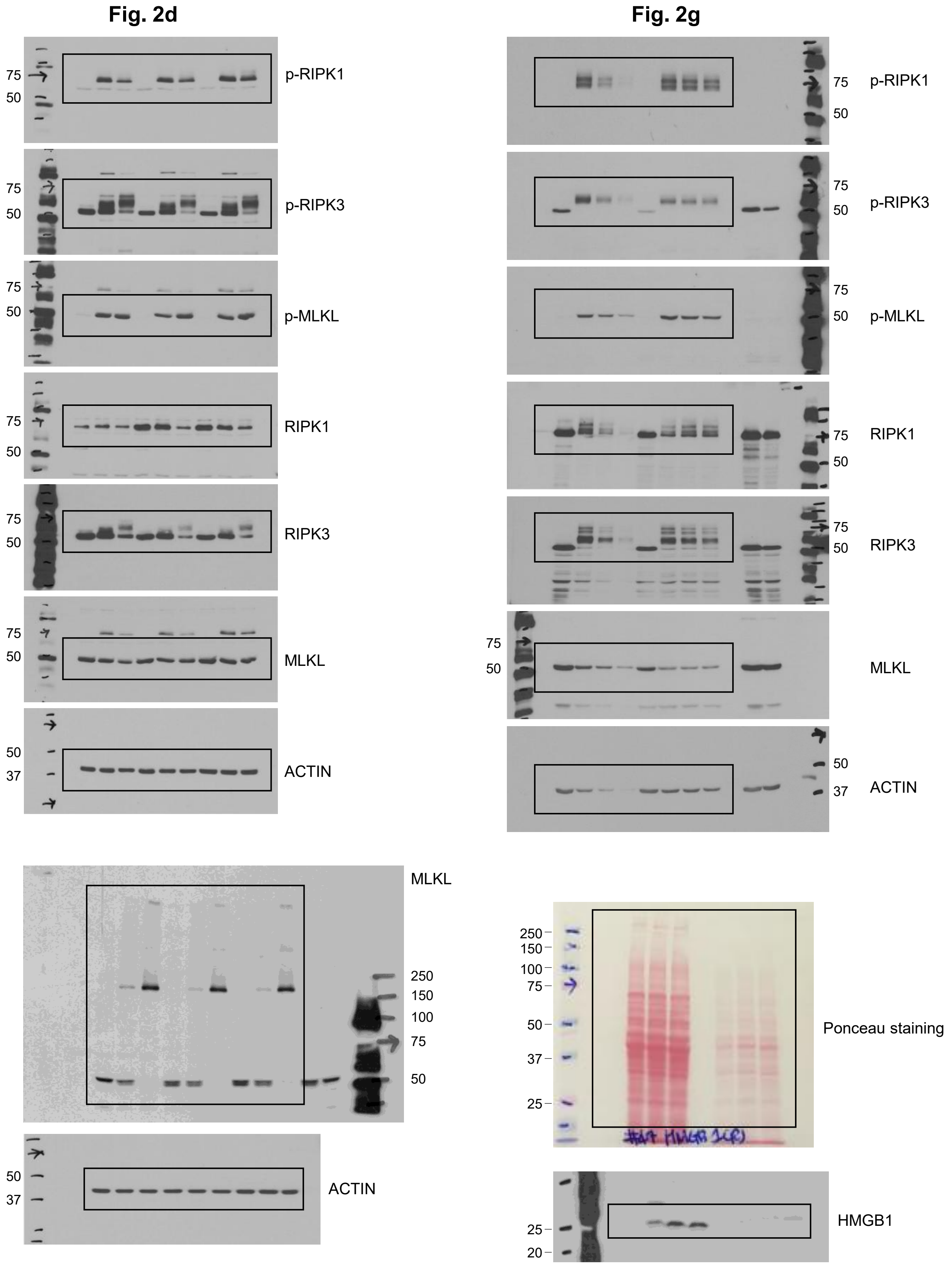
Fig. 2d
Fig. 2g
p-RIPK1
p-RIPK3
p-MLKL
RIPK1
RIPK3
MLKL
ACTIN
Ponceau staining
HMGB1
250
150
100
75
50
37
25
20

Fig. 3.

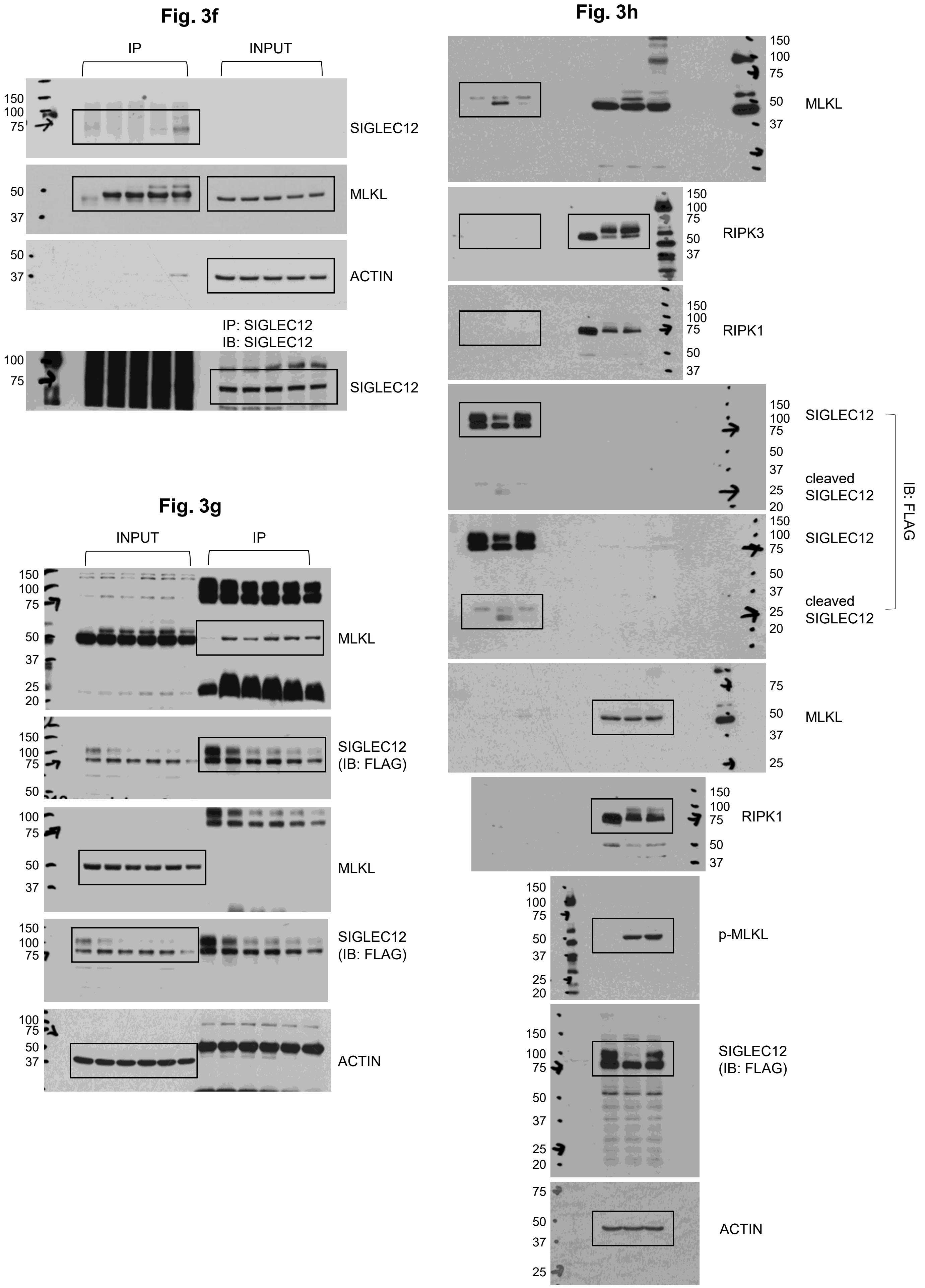

Fig. 3f
IP
INPUT
SIGLEC12
MLKL
ACTIN
IP: SIGLEC12
IB: SIGLEC12
SIGLEC12
Fig. 3g
INPUT
IP
MLKL
SIGLEC12
(IB: FLAG)
MLKL
SIGLEC12
(IB: FLAG)
ACTIN
Fig. 3h
MLKL
RIPK3
RIPK1
SIGLEC12
cleaved
SIGLEC12
SIGLEC12
cleaved
SIGLEC12
IB: FLAG
MLKL
RIPK1
p-MLKL
SIGLEC12
(IB: FLAG)
ACTIN
150
100
75
50
37
25
20

Fig. 4.

Fig. 4a

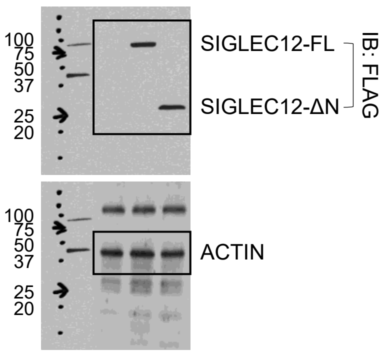

Fig. 4b

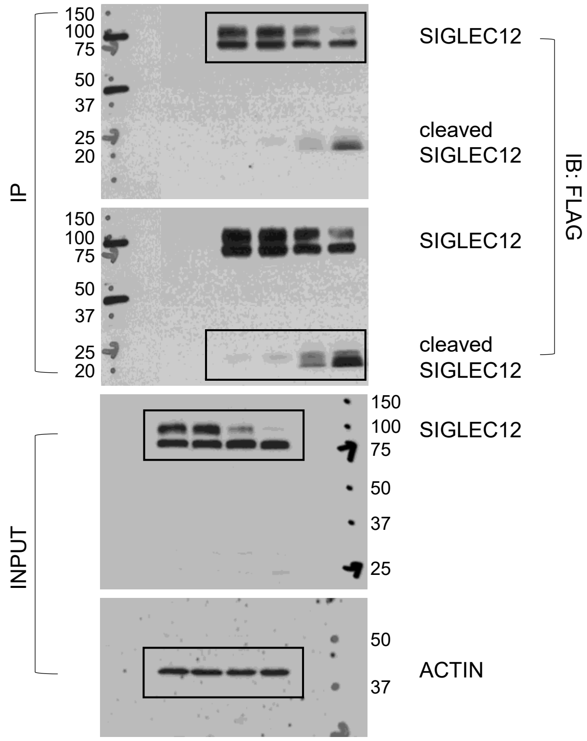

Fig. 4c

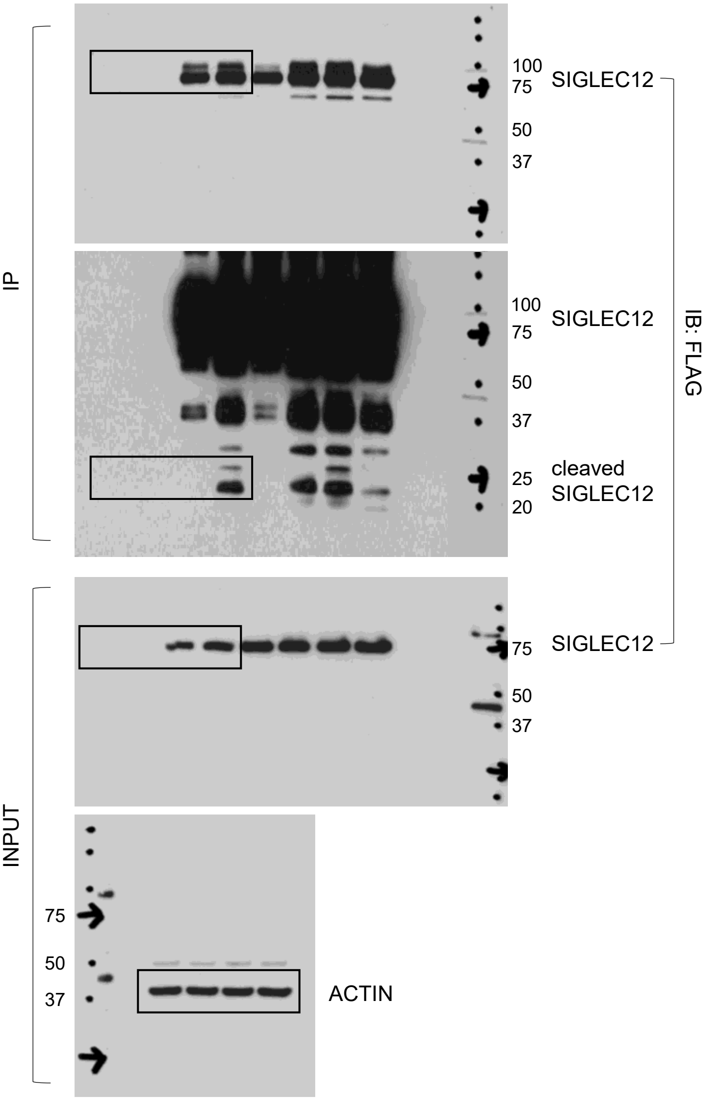

Fig. 4f

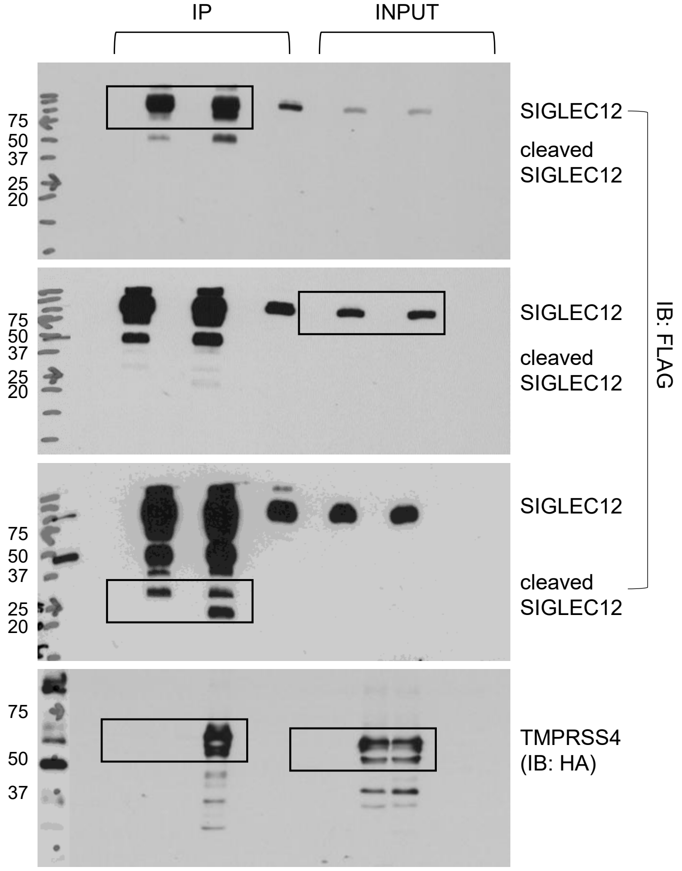

Fig. 4.

Fig. 4g

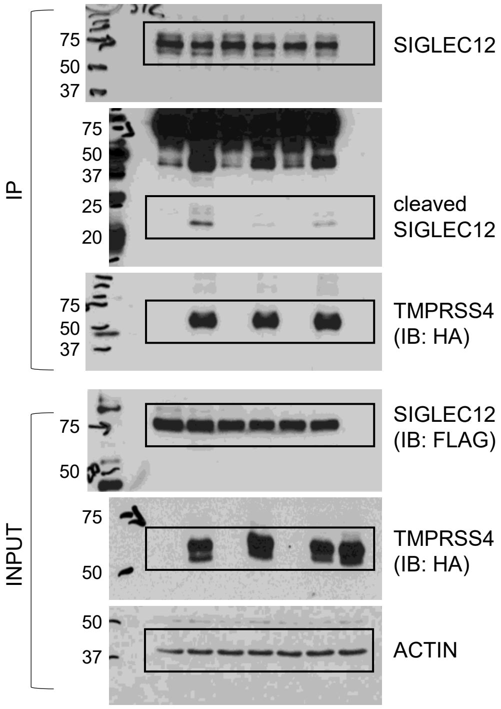

Fig. 4i

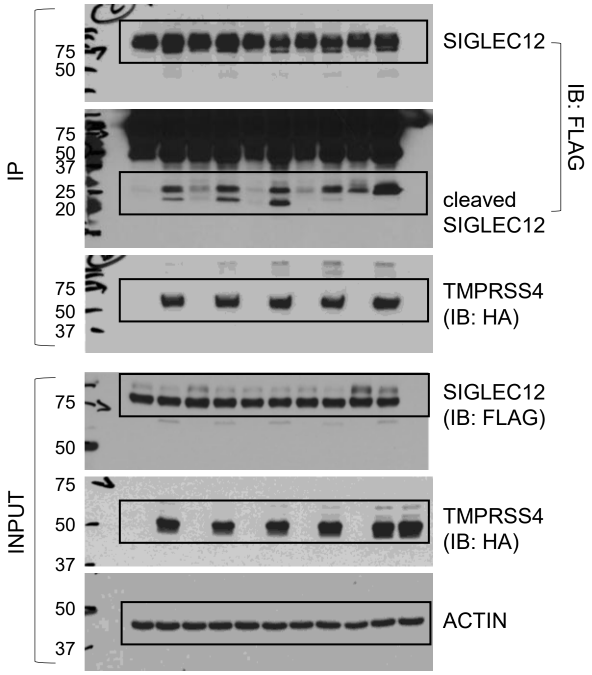

Extended Data Fig. 1.

Extended Data Fig. 1a

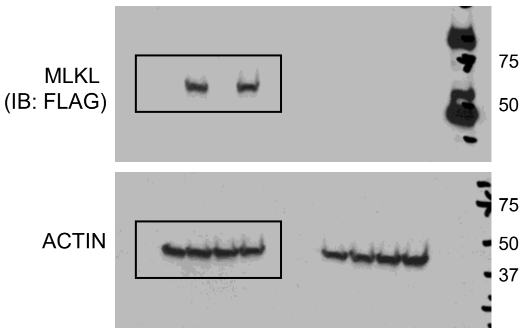

Extended Data Fig. 1j

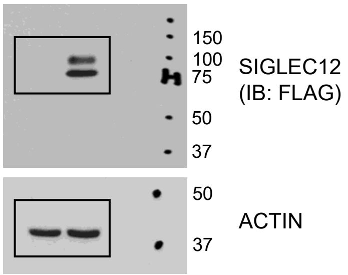

Extended Data Fig. 1b

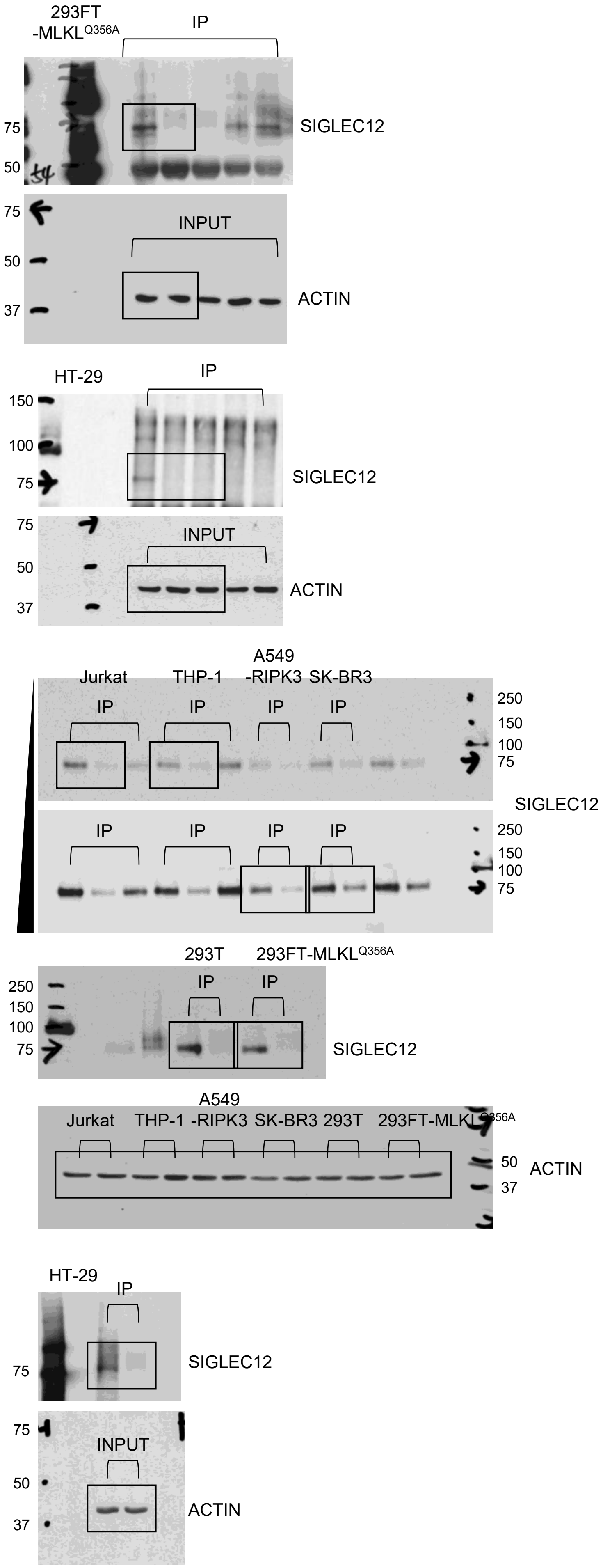

Extended Data Fig. 4.

Extended Data Fig. 4e

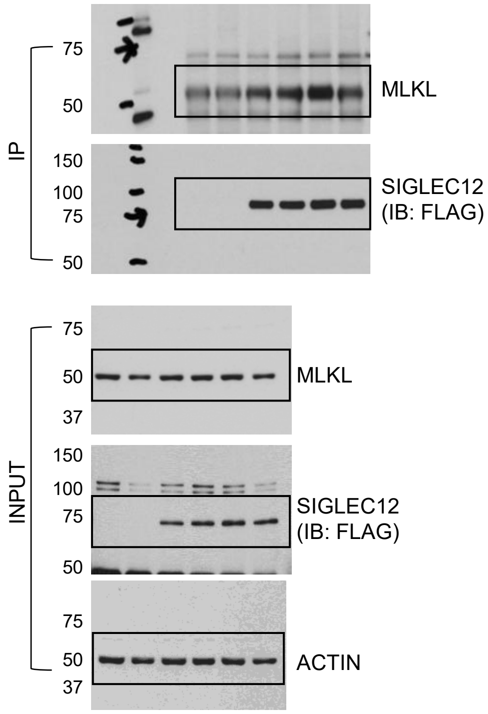

Extended Data Fig. 4f

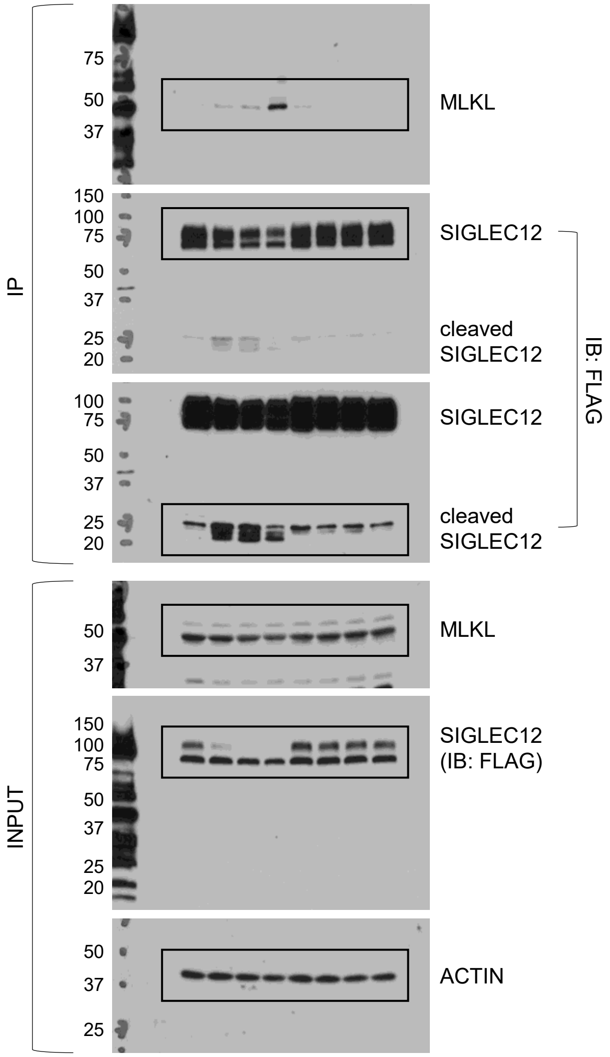

Extended Data Fig. 4h

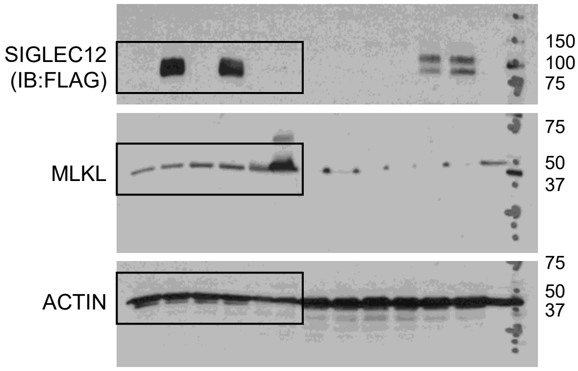

Extended Data Fig. 5.

Extended Data Fig. 5a

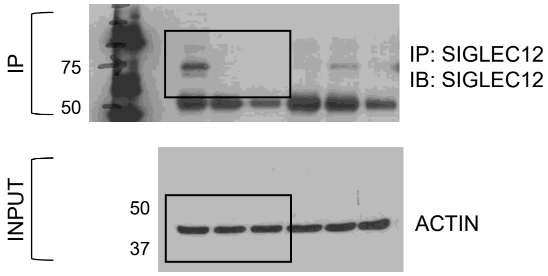

Extended Data Fig. 6.

Extended Data Fig. 6d

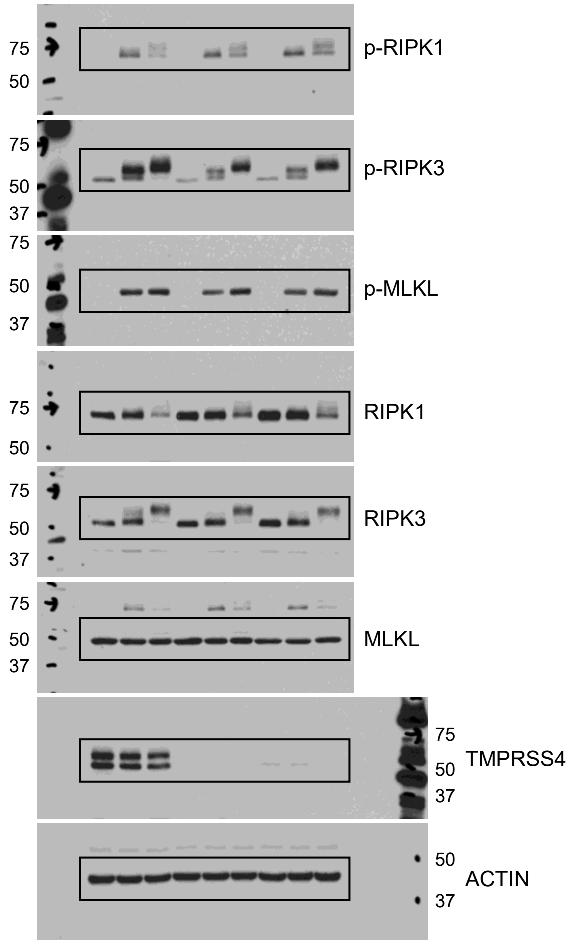

Extended Data Fig. 6e

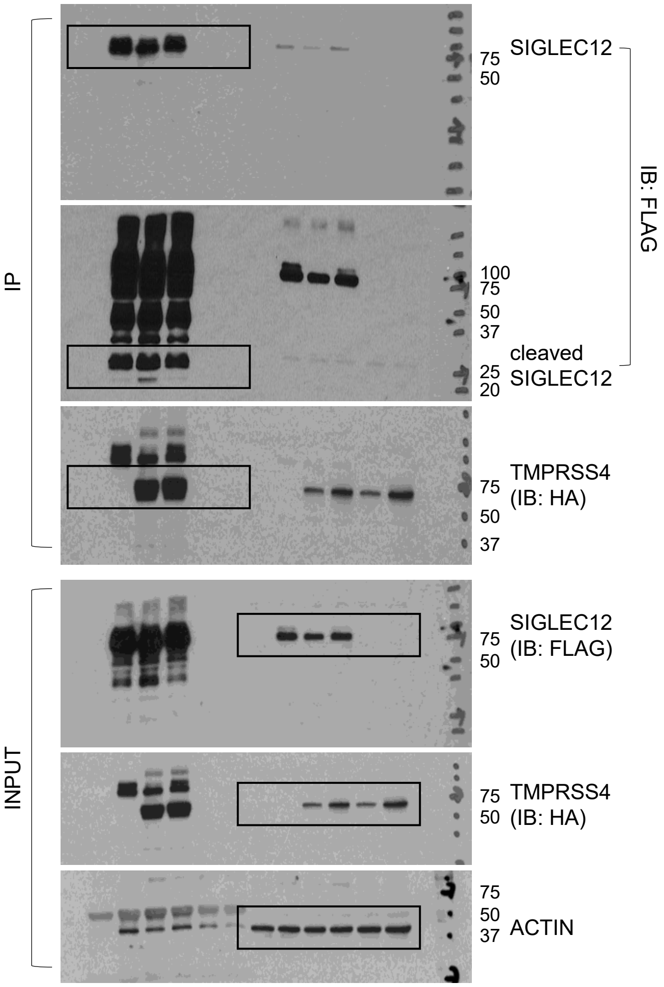

Extended Data Fig. 6.

Extended Data Fig. 6g

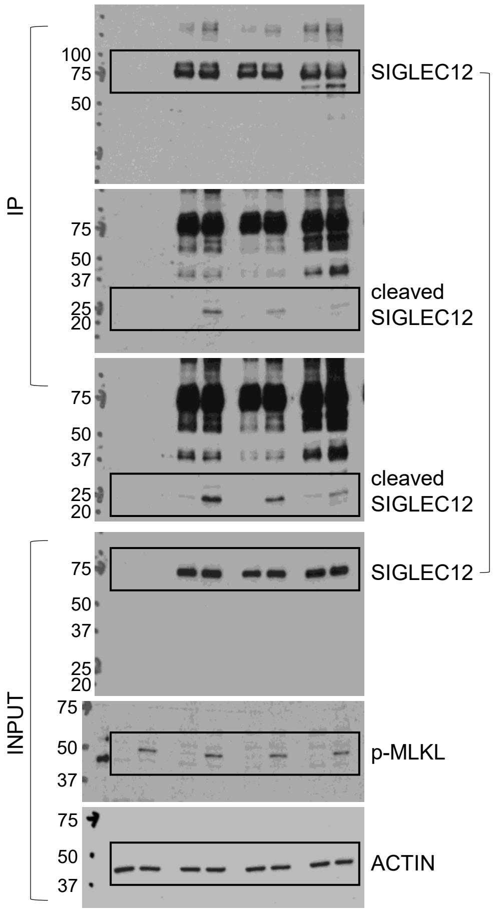

Extended Data Fig. 6j

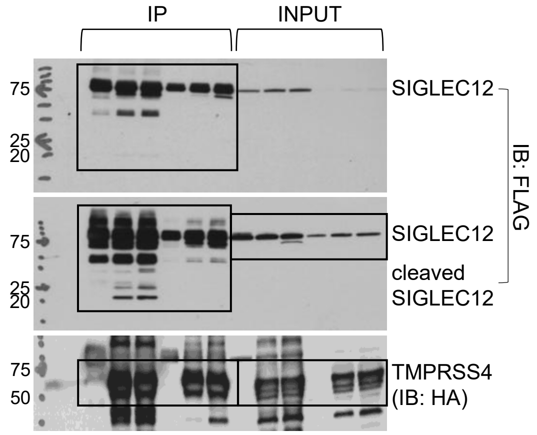

Extended Data Fig. 6k

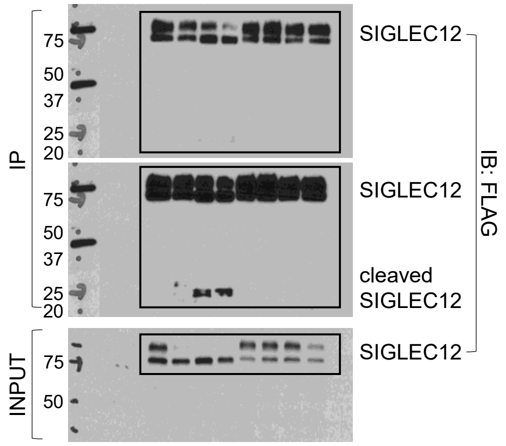

Supplement: Supplementary file 1 — Supplementary Fig. 1 [file 41586_2025_9741_MOESM1_ESM.pdf]
