# Supplementary material for: SIGLEC12 mediates plasma membrane rupture during necroptotic cell death
Source: Nature. 2025 Nov 12;649(8096):460–6. doi: 10.1038/s41586-025-09741-1 (PMC12779560; doi:10.1038/s41586-025-09741-1)
Supplement: Supplementary file 2 — Reporting Summary [file 41586_2025_9741_MOESM2_ESM.pdf]

nature research

Corresponding author(s): Ayaz Najafov

Last updated by author(s): Sep 29, 2025

Reporting Summary

Nature Research wishes to improve the reproducibility of the work that we publish. This form provides structure for consistency and transparency in reporting. For further information on Nature Research policies, see Authors & Referees and the Editorial Policy Checklist.

Statistics

For all statistical analyses, confirm that the following items are present in the figure legend, table legend, main text, or Methods section.

n/a	Confirmed	
☐	☒	The exact sample size (*n*) for each experimental group/condition, given as a discrete number and unit of measurement
☐	☒	A statement on whether measurements were taken from distinct samples or whether the same sample was measured repeatedly
☐	☒	The statistical test(s) used AND whether they are one- or two-sided<br>*Only common tests should be described solely by name; describe more complex techniques in the Methods section.*
☐	☒	A description of all covariates tested
☐	☒	A description of any assumptions or corrections, such as tests of normality and adjustment for multiple comparisons
☐	☒	A full description of the statistical parameters including central tendency (e.g. means) or other basic estimates (e.g. regression coefficient) AND variation (e.g. standard deviation) or associated estimates of uncertainty (e.g. confidence intervals)
☐	☒	For null hypothesis testing, the test statistic (e.g. *F*, *t*, *r*) with confidence intervals, effect sizes, degrees of freedom and *P* value noted<br>*Give P values as exact values whenever suitable.*
☒	☐	For Bayesian analysis, information on the choice of priors and Markov chain Monte Carlo settings
☒	☐	For hierarchical and complex designs, identification of the appropriate level for tests and full reporting of outcomes
☒	☐	Estimates of effect sizes (e.g. Cohen's *d*, Pearson's *r*), indicating how they were calculated

Our web collection on statistics for biologists contains articles on many of the points above.

Software and code

Policy information about availability of computer code

Data collection	Nikon NIS-Elements™ image acquisition software for microscopy, 21 CFR Part 11 Software Module for QuantStudio™ 6 & 7 Systems for qRT-PCR, My Image Garden™ software of Canon MX452™ scanner for western blot film and crystal violet-stained culture plate scanning, Sequencing Analysis Software of ABI3730xl™ DNA analyzer for sequencing data collection, SkanIt™ v7.0.2 for Varioskan LUX™ plate reader (Thermo) for luminescence data collection, Incucyte® S3-C2 software for cell death data collection.
Data analysis	R Studio v2025.09, Fiji/ImageJ v1.54p, PhenoChart™ v1.1.0, Graph Pad Prism™ v10.2, Adobe Illustrator™ v28.6, Adobe Photoshop™ v24.1, Microsoft Excel™ (2024), Clustal Omega (2024), JalView v2.11.5.0.

For manuscripts utilizing custom algorithms or software that are central to the research but not yet described in published literature, software must be made available to editors/reviewers. We strongly encourage code deposition in a community repository (e.g. GitHub). See the Nature Research guidelines for submitting code & software for further information.

Data

Policy information about availability of data

All manuscripts must include a data availability statement. This statement should provide the following information, where applicable:
- Accession codes, unique identifiers, or web links for publicly available datasets
- A list of figures that have associated raw data
- A description of any restrictions on data availability

All data reported in this manuscript will be made public upon request. Data from NCBI dbSNP database and ProteinAtlas database were used in this study.

Field-specific reporting

Please select the one below that is the best fit for your research. If you are not sure, read the appropriate sections before making your selection.

☒ Life sciences ☐ Behavioural & social sciences ☐ Ecological, evolutionary & environmental sciences

For a reference copy of the document with all sections, see nature.com/documents/nr-reporting-summary-flat.pdf

Life sciences study design

All studies must disclose on these points even when the disclosure is negative.

Sample size	To ensure an adequate power of the statistical testing, we perform special analyses prior to running the experiments, to calculate how large an n is required. The null hypothesis (H0) = no difference between the untreated versus treated, while the alternative hypothesis (H1) = outcome where there is a difference. The power calculation is: power = P (reject Ho | H1 is true) = P (accept H1 | H1 is true). We estimate that the probability to obtain a difference between the groups is ~95% and that there is only 5% H0 is true. n = logβ/logp, where β is the probability of committing a Type II error (0.05) and p is the proportion of the animals in the group that are not affected by the treatments. To obtain at least 50% of mice altered by the treatment in one group, we will need: n=log(0.05)/log(0.5) = 4.32 ~ 5 mice.
Data exclusions	No data was excluded.
Replication	Each experiment in the figures was independently repeated at least three times.
Randomization	*Describe how samples/organisms/participants were allocated into experimental groups. If allocation was not random, describe how covariates were controlled OR if this is not relevant to your study, explain why.*
Blinding	*Describe whether the investigators were blinded to group allocation during data collection and/or analysis. If blinding was not possible, describe why OR explain why blinding was not relevant to your study.*

Reporting for specific materials, systems and methods

We require information from authors about some types of materials, experimental systems and methods used in many studies. Here, indicate whether each material, system or method listed is relevant to your study. If you are not sure if a list item applies to your research, read the appropriate section before selecting a response.

Materials & experimental systems

n/a	Involved in the study	
☐	☒	Antibodies
☐	☒	Eukaryotic cell lines
☒	☐	Palaeontology
☒	☐	Animals and other organisms
☐	☒	Human research participants
☒	☐	Clinical data

Methods

n/a	Involved in the study	
☒	☐	ChIP-seq
☐	☒	Flow cytometry
☒	☐	MRI-based neuroimaging

Antibodies

Antibodies used	The following antibodies were used in this study: RIPK1 (Cell Signaling Technology, #3493); p-hRIPK1 (Cell Signaling Technology, #65746); hRIPK3 (Cell Signaling Technology, #13526); p-hRIPK3 (Cell Signaling Technology, #93654); hMLKL (Cell Signaling Technology, #14993; Abcam, ab183770; Thermo Fisher Scientific, PA5-34733); p-hMLKL (Cell Signaling Technology, #91689); Anti-SIGLEC12 (Boster, A10550-1; Invitrogen, PA5-110369; Invitrogen, PA5-31457); TMPRSS4 (Cell Signaling Technology, #84382); Anti-HMGB1 (Cell Signaling Technology, #6893); Anti-β-Actin (Sigma-Aldrich, A5316); Anti-Flag (Sigma-Aldrich, F1804); Anti-Flag-HRP (Cell Signaling Technology, #86861); Anti-mouse IgG (Cell Signaling Technology, #7074); Anti-rabbit IgG (Cell Signaling Technology, #7076), Anti-mouse IgG (H+L), F(ab')2 Fragment (Alexa Fluor® 488 Conjugate) (Cell Signaling Technology, #4408). All primary antibodies were used at 1:2000 dilution. All secondary antibodies were used at 1:5000 dilution.
Validation	The antibodies were validated by their vendors using western blotting. Validation data is available on the vendor websites.

Eukaryotic cell lines

Policy information about cell lines

Cell line source(s)	HEK293T cells (ATCC), HT-29 cells (ATCC), HeLa cells (ATCC), Jurkat cells (ATCC), SK-BR-3 cells (ATCC), THP-1 cells (ATCC), A549 cells (ATCC), MEF cells (generated in the Najafov lab).

Authentication	Cells were authenticated by genotyping and western blotting.
Mycoplasma contamination	All cell lines were regularly tested for mycoplasma contamination using Lonza's MycoAlert Kit and tested negative for mycoplasma.
Commonly misidentified lines (See ICLAC register)	HT-29 cells are present in this database. Rationale: These cells are a potent necroptosis model with a high rate of CRISPR. HT-29 cells were obtained from ATCC and stocks were carefully maintained with new frozen batch thawed every 6 weeks. The cells were identified by their distinct morphology and sensitivity to TSE-induced necroptosis.

Human research participants

Policy information about studies involving human research participants

Population characteristics	*Describe the covariate-relevant population characteristics of the human research participants (e.g. age, gender, genotypic information, past and current diagnosis and treatment categories). If you filled out the behavioural & social sciences study design questions and have nothing to add here, write "See above."*
Recruitment	*Describe how participants were recruited. Outline any potential self-selection bias or other biases that may be present and how these are likely to impact results.*
Ethics oversight	De-identified human benign (normal) lung and colon and lung and colon adenocarcinoma tumor specimens were obtained from the University of Texas Southwestern Tissue Management Shared Resource. Patients were enrolled and consented to an Institutional Review Board-approved protocol.

Note that full information on the approval of the study protocol must also be provided in the manuscript.

Flow Cytometry

Plots

Confirm that:

☐ The axis labels state the marker and fluorochrome used (e.g. CD4-FITC).

☐ The axis scales are clearly visible. Include numbers along axes only for bottom left plot of group (a 'group' is an analysis of identical markers).

☐ All plots are contour plots with outliers or pseudocolor plots.

☐ A numerical value for number of cells or percentage (with statistics) is provided.

Methodology

Sample preparation	*Describe the sample preparation, detailing the biological source of the cells and any tissue processing steps used.*
Instrument	*Identify the instrument used for data collection, specifying make and model number.*
Software	*Describe the software used to collect and analyze the flow cytometry data. For custom code that has been deposited into a community repository, provide accession details.*
Cell population abundance	*Describe the abundance of the relevant cell populations within post-sort fractions, providing details on the purity of the samples and how it was determined.*
Gating strategy	*Describe the gating strategy used for all relevant experiments, specifying the preliminary FSC/SSC gates of the starting cell population, indicating where boundaries between "positive" and "negative" staining cell populations are defined.*

☐ Tick this box to confirm that a figure exemplifying the gating strategy is provided in the Supplementary Information.
