# Supplementary material for: SIGLEC12 mediates plasma membrane rupture during necroptotic cell death
Source: Nature. 2025 Nov 12;649(8096):460–6. doi: 10.1038/s41586-025-09741-1 (PMC12779560; doi:10.1038/s41586-025-09741-1)
Supplement: Supplementary file 3 — Time-lapse live-cell imaging confocal microscopy of PMR in HT-29-shGFP stable cell line during necroptosis (bright-field and CellToxGreen channels). Stills are shown in Fig. 2d (left panel). HT-29-shGFP stable shRNA-mediated knockdown cell line was treated with TSE for 24 h, and cell death was assessed using CellToxGreen and time-lapse fluorescence confocal microscopy. A merge of bright-field and green fluorescence channels is shown. Representative of three independent experiments. [file 41586_2025_9741_MOESM3_ESM.pptx]

## Slide 1
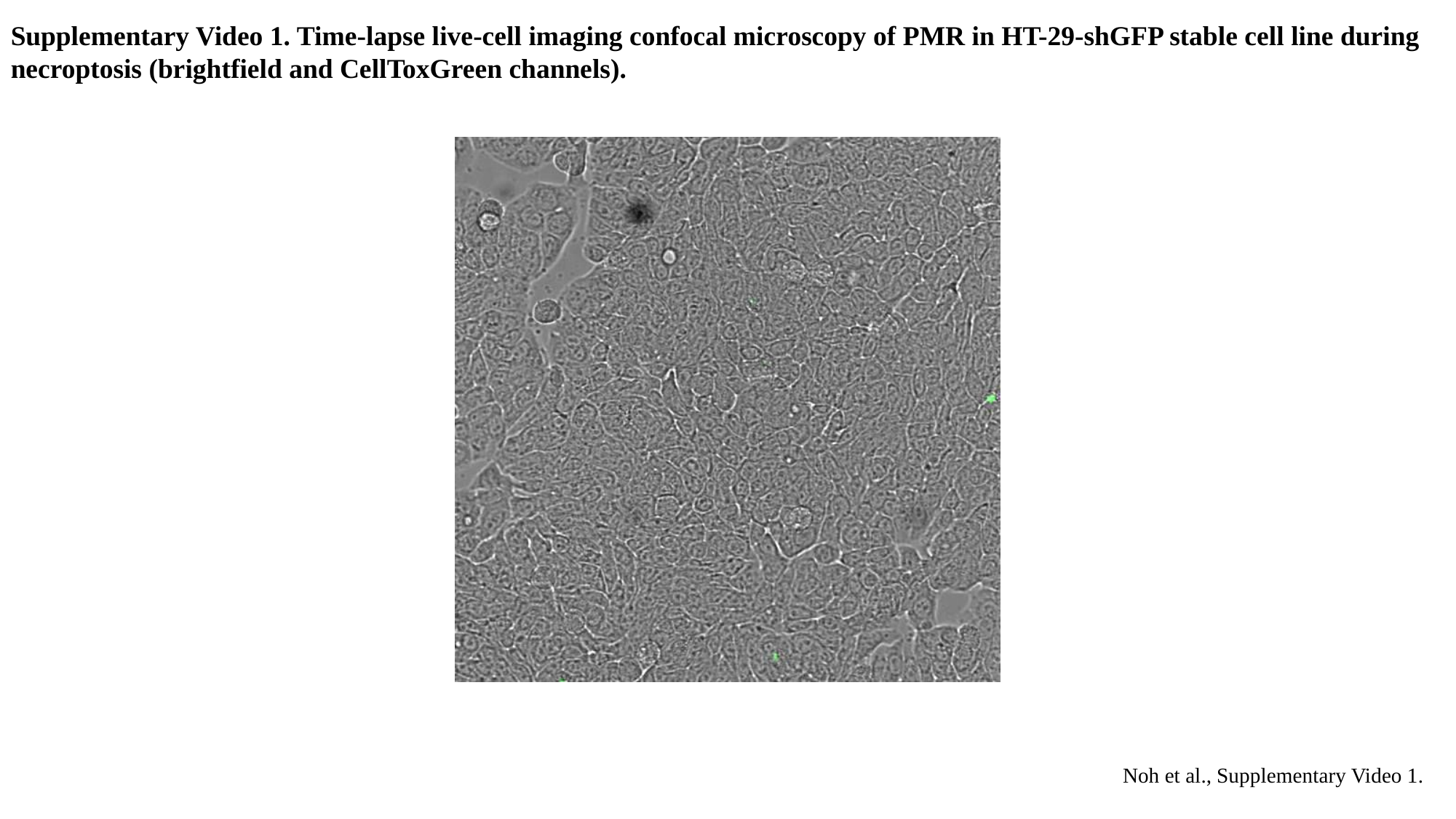

Supplementary Video 1. Time-lapse live-cell imaging confocal microscopy of PMR in HT-29-shGFP stable cell line during necroptosis (brightfield and CellToxGreen channels).
Noh et al., Supplementary Video 1.
